# Additive Manufacturing of High‐Performance Polymers for Extreme Environments

**DOI:** 10.1002/advs.77371

**Published:** 2026-10-14

**Authors:** Md Tofazzal Hossain, Mohammed Intishar Nur, Md Shajedul Hoque Thakur, Indranil Dey, Md Mesbahul Maruf, Md Jahid Hasan, Suryasarathi Bose, Muhammad M. Rahman

**Affiliations:** ^1^ Department of Mechanical and Aerospace Engineering University of Houston Texas USA; ^2^ Department of Materials Science and NanoEngineering Rice University Texas USA; ^3^ Department of Materials Engineering Indian Institute of Science Bangalore India; ^4^ Department of Biosystems Engineering Auburn University Alabama USA

**Keywords:** additive manufacturing, direct ink writing, fused filament fabrication, high‐performance polymers, selective laser sintering, thermoplastics

## Abstract

Additive manufacturing (AM) of high‐performance polymers (HPPs) has gained significant research interest due to its potential for applications in extreme environments, including aerospace, automotive, marine, and defense sectors. Components in these applications are often subjected to prolonged exposure to high temperatures, high mechanical loads, and chemically aggressive environments. HPPs are well‐suited to such demanding conditions because of their high mechanical properties, thermal stability, flame resistance, and chemical durability. However, the inherent molecular and structural characteristics that provide these properties also pose notable challenges during AM of HPPs. In particular, the effective processing and fabrication of HPPs using AM techniques are limited by their rigid molecular structure, high melting temperatures and viscosities, and complex crystallization behavior. This review focuses on the processing and utilization of HPPs using commercially available AM techniques. Particular emphasis is placed on the relationships among polymer chemistry, rheology, thermal history, crystallinity, process‐induced defects, and mechanical performance. Recent applications of additively manufactured HPPs are critically evaluated. The review also compares qualification requirements, environmental durability, and technology readiness with those of conventionally manufactured components. Finally, unresolved challenges and future research priorities are identified to enable reliable, scalable, and certification‐ready additively manufactured HPP components for extreme‐service environments.

## Graphical abstract

Additive manufacturing of high‐performance polymers for extreme environments is critically reviewed through an integrated chemistry‐processing‐structure‐property‐qualification framework. The review compares fused filament fabrication, selective laser sintering, and direct ink writing, linking rheology and thermal history to crystallization, defect formation, and performance. Key governing principles, qualification needs, and deployment readiness are outlined, along with future priorities for reliable, scalable, certification‐ready components.

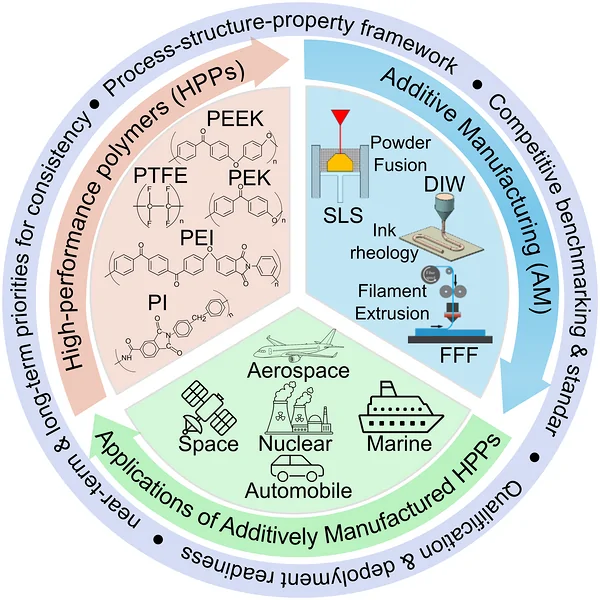

## 1 Introduction

Engineering components operating in aerospace, automotive, electronics, oil and gas, defense, and marine sectors are often exposed to extreme environments. Representative conditions include elevated temperatures above 125°C and pressures above 200 bar, although the relevant thresholds are application‐dependent [1]. Extreme service may also involve cyclic mechanical loading, time‐dependent deformation, impact, intense electromagnetic fields, cryogenic temperatures, concentrated salts, oxidizing agents, and strong acidic or alkaline solutions [2]. These combined thermal, mechanical, physical, and chemical exposures can degrade structural integrity and material properties. Consequently, ensuring the long‐term durability and reliability of components in such extreme conditions necessitates the development of advanced high‐performance materials. Among these, high‐performance polymers (HPPs) are promising due to their exceptional material properties, including high strength‐to‐weight ratios, thermal stability, and chemical resistance [1].

The recent advancements in HPPs and their tailored material properties make them highly attractive for a range of critical applications, including extreme environments. Commonly used HPPs include polyetherimide (PEI), poly(ether ether ketone) (PEEK), poly(etherketoneketone) (PEKK), polyetherketone (PEK), poly(tetrafluoroethylene) (PTFE), polyamide‐imide (PAI), poly(vinylidene fluoride) (PVDF), poly(ethersulfone) (PES), polyimides (PI), polysulfone (PSU), liquid crystalline polymers (LCP), and poly(phenylene sulfide) (PPS). Typically, these polymers maintain dimensional stability at operating temperatures above 150°C, making them suitable for elevated‐temperature applications [3]. Notably, these polymers have thermal degradation temperatures exceeding ∼400°C [4, 5, 6, 7] and can withstand prolonged exposure to temperatures above 177°C for over 10,000 h [8, 9]. Additional benchmarks of HPPs include minimal weight‐loss rates at elevated temperatures, such as 0.05 wt.% per hour at 450°C, and a slight mass loss (∼5%), measured by thermogravimetric analysis (TGA) [9, 10]. The high performance of these polymers is primarily attributed to their long and rigid molecular structures, which result in high glass transition temperatures (T_g_) typically above 200°C and retention of mechanical performance at elevated service temperatures [9, 10]. In addition, many of these HPPs exhibit very low surface friction and high hydrophobicity. These outstanding tribological characteristics reduce surface wear, allowing for reliable relative motion even under elevated temperatures and humidity.

In contrast to conventional polymers, HPPs are predominantly aromatic in their molecular chain structure [10] and are engineered to withstand extreme conditions. In recent years, significant usage of HPPs has been observed for various functional and structural components, especially in aircraft applications. For instance, PI and its composites are used for aircraft engine nacelles due to their outstanding thermal stability and flame‐retardant properties, providing a protective layer for engine compartments at elevated temperatures. PEEK and its composites have been utilized as alternatives to metals in applications such as landing gear components, fuselage frames, fuel tanks, and engine mounts. PEI and other flame‐resistant polymers are used in the aircraft tail assembly, including stabilizers, trim tabs, and elevators, which require lightweight yet strong materials to ensure high structural stability and smooth aerodynamic control. Furthermore, HPPs are used in the interior compartments, including the cockpit, air ducts, seat framework, wall panels, and armrest caps. Overall, the recent use of these HPPs, with improved performance, efficiency, and safety, underscores their suitability for demanding environments [11].

High‐performance polymer‐based components are typically fabricated using conventional manufacturing techniques, such as compression molding, injection molding, tooling, and extrusion processes. While these conventional methods offer efficiency in bulk manufacturing, they are often limited in their ability to fabricate complex geometries, require costly tooling, and lack design flexibility. Consequently, additive manufacturing (AM) emerges as a preferred alternative for specialized and intricate applications, offering enhanced customization, compatibility with a wide range of materials, and geometric design flexibility. AM fabricates 3D objects by layer‐by‐layer deposition of desired materials, significantly reducing material waste and enabling fast prototyping. Additionally, AM does not require intricate tooling, offers design freedom, and is capable of net‐shape fabrication. The recent advancements in HPPs have also broadened the scope of applications for polymer‐based AM techniques, extending their use to produce architected structures for extreme environments. For example, the United Launch Alliance (ULA) utilized AM techniques to print ducting system components using HPPs that replaced many expensive metal counterparts [12]. The National Aeronautics and Space Administration (NASA) has also deployed AM techniques for various critical parts in its space stations. These developments highlight the growing versatility and crucial importance of polymer‐based AM techniques in modern manufacturing and engineering sectors [13, 14]. However, processing HPPs in AM remains challenging due to factors such as chemical complexities in molecular formulation, high melting and processing temperatures, inadequate flow behavior, molecular degradation, insufficient interlayer adhesion, warping, and anisotropic mechanical properties in 3D‐printed parts [15, 16]. A hierarchical classification of various thermoplastic polymers based on their material integrity and stability under chemical, thermal, and mechanical conditions, with suitable AM techniques and various applications, is presented in Figure 1. As shown in the figure, fused filament fabrication (FFF), selective laser sintering (SLS), and direct ink writing (DIW)‐based AM techniques have demonstrated adaptability for HPPs through the optimization of chemical formulations and operating conditions [17].

**FIGURE 1:**
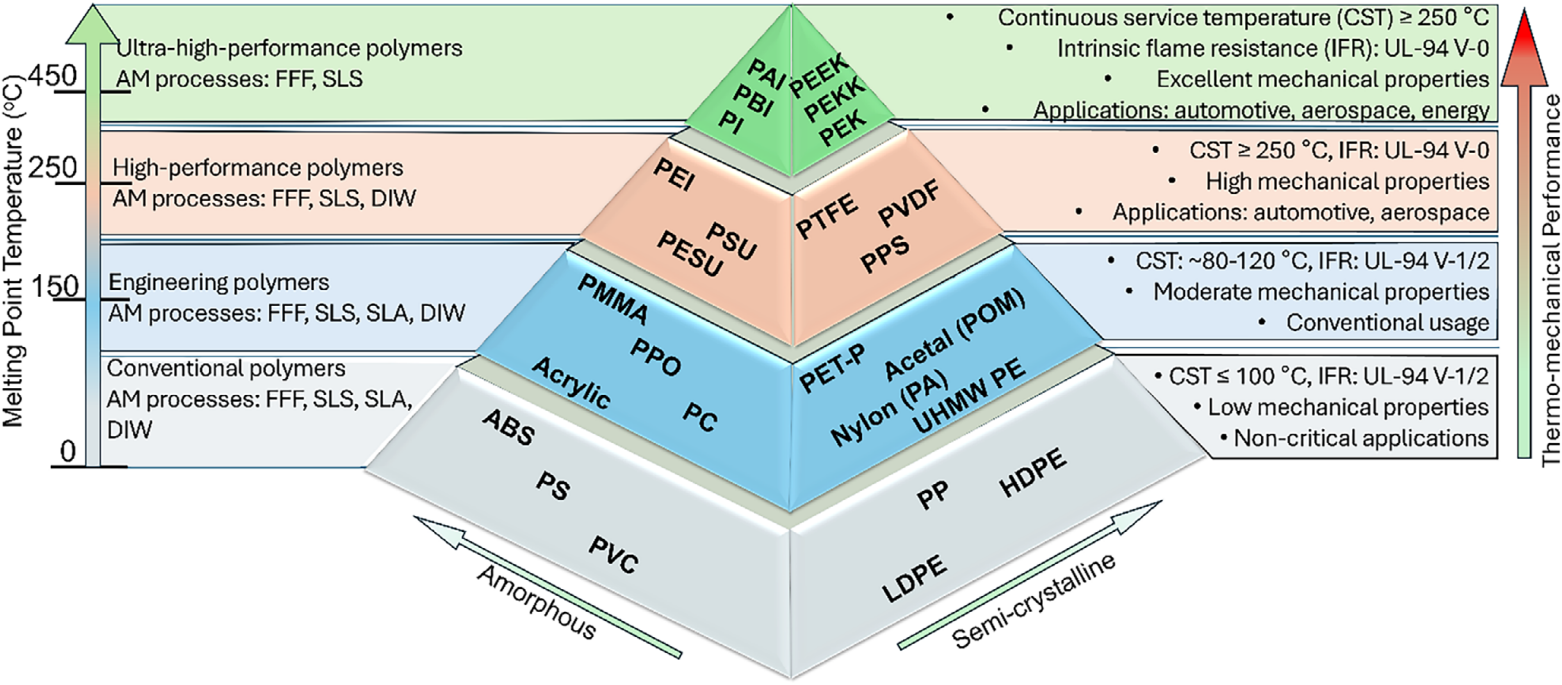
Hierarchical classification of polymers according to melting temperature, chemical resistance, flame retardancy, and thermomechanical performance. Polymer classes are arranged from conventional polymers to ultra‐high‐performance polymers, with representative additive manufacturing (AM) processes indicated for each class. The pyramid illustrates increasing structural stability and suitability for extreme‐environment applications [17].

This review provides a comprehensive overview of AM techniques for HPPs, highlighting recent advancements in research and development. Despite growing interest in AM of HPPs since the early 2000s, several review articles have critically explored AM of HPPs; however, they are often limited to specific polymer classes, AM processes, and optimization strategies. For instance, Das et al. [15] critically reviewed material extrusion and powder‐bed‐fusion technologies, whereas Magri et al. [18] focused primarily on the FFF technique and process optimization for HPPs. Similarly, Simunec et al. [19] comprehensively reviewed a limited number of HPP families (poly(aryletherketone), PPS, and PEI‐based systems) and polymer blend strategies, focusing on melt rheology, printability, and crystallization behavior. Vidakis et al. [20] similarly emphasized material extrusion, polymer chemistry, melt rheology, process optimization, and mechanical performance. Collectively, these works have substantially addressed specific processing techniques and material systems; however, an integrated and strategic review that systematically connects unified process‐structure‐property relationships with polymer chemistry, rheology, thermal history, qualification, performance benchmarking, technology readiness, and behavior under extreme service conditions remains limited. This literature gap motivates a service‐oriented assessment linking the intrinsic properties of HPPs to AM processing requirements and retained performance in extreme environments. Accordingly, this review examines recent developments, technical challenges, and emerging opportunities across these interconnected themes.

## 2 Representative High‐Performance Polymer Chemistry and Properties

Realizing the full potential of AM for HPPs requires a clear understanding of the polymer structures and thermomechanical properties that govern both processability and in‐service performance. The aromatic backbones, rigid chain architectures, and strong intermolecular interactions responsible for the exceptional performance of HPPs in extreme environments also control their melt rheology, thermal transitions, crystallization behavior, and printability. Table 1 summarizes these key thermal and mechanical properties across representative HPPs, providing a basis for comparing their suitability for different extreme‐environment applications [21, 22, 23, 24, 25].

**TABLE 1:** Comparative thermal and mechanical properties and molecular structures of representative high‐performance polymers.

Polymers	Molecular structure	Glass transition temperature, T_g_ (°C)	Melting temperature, T_m_ (°C)	Processing window (°C)	Mechanical properties
PEI [21]	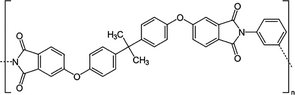	215–230	—	320–370	UTS: 100–105 MPa E (flexural): ∼3.2 GPa Notched Izod Impact strength: 50 – 60 J m–^1^
PEEK [21]	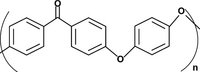	143	343	360–400	UTS: 90–100 MPa E (flexural): 3.5 – 4.0 GPa Notched Izod Impact strength: 80–90 J m–^1^
PEKK [21]	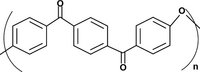	150–165	305–360	340–400	UTS: 90–100 MPa E (flexural): 3.5 – 4.0 GPa Notched Izod Impact strength: 80–90 J m–^1^
PEK [22]	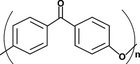	165	373	400	UTS: 105 MPa E (tensile): 4.0 GPa Elongation (tensile): 5%
PI [23]	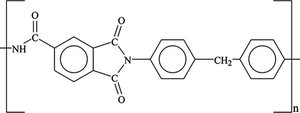	195–250	210–400	350–370	UTS: 65–125 MPa E (tensile): 1–5.6 GPa Notched Impact strength: 5.3 kJ m–^2^
PTFE [24, 25]	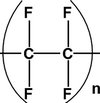	—	317–337	379–429	UTS: 21–34 MPa E (flexure): 345 – 620 MPa Notched Izod Impact strength: 80–320 J m–^1^

PEI is one of the most widely used HPPs owing to its ease of processability, excellent mechanical properties, and high‐temperature stability. ULTEM 9085 and ULTEM 1010, two popular types of PEI developed by General Electric's (GE) Plastics Division in the early 1980s, are now commercially graded for a wide range of demanding applications. PEI exhibits excellent material properties, primarily owing to two strong functional groups in its molecular structure: ether (‐O‐) and imide (‐CO‐N‐CO‐) (Table 1). While the flexible ether group provides excellent material toughness, the aromatic imide groups promote thermal stability and mechanical strength through their rigid molecular backbone [26]. Additionally, PEI has intrinsic flame resistance due to its rigid chemical composition. This feature, combined with its low smoke production and high limiting oxygen index (LOI), promotes self‐extinguishing behavior and minimizes melt dripping, making it suitable for high‐temperature applications [27].

Polyaryletherketone (PAEK) is a group of aromatic and semicrystalline HPPs consisting of two principal functional groups: ether (‐O‐) and ketone (–CO–). These HPPs, led by PEEK, PEKK, and PEK, have gained attention in advanced manufacturing technologies because of their excellent material properties (Table 1). Like PEI, the rigid aromatic structure and presence of two functional groups make them stable at high temperatures and in strong chemical environments. The number of ether and ketone groups, molecular arrangement, and the molecular weight of the polymer chains primarily govern the properties of these HPPs. For instance, the higher ether content in the backbone enhances toughness and flexibility but reduces their rigidity, mechanical strength, and thermal stability. Conversely, a higher concentration of ketone groups leads to increased stiffness, strength, T_m_, and T_g_ [28]. Among these PAEK polymers, PEEK is widely known for its remarkable chemical inertness, continuous stability at high temperatures (∼250°C), and short‐term stability at temperatures exceeding 400°C [17, 29, 30]. PEKK is also graded for multifunctional applications. For example, along with excellent mechanical properties, Kepstan, the most widely used PEKK, exhibits strong inherent flame retardancy and emits minimal smoke and toxic gases, making it a promising material for chemical and aerospace applications [31]. On the other hand, PEK is often insoluble in various solvents, highlighting its strong chemical resilience in different harsh environments. Additionally, the morphology and performance of the PEK polymer are significantly influenced by its thermal processing conditions. These variances in morphology and crystallinity, resulting from varying cooling rates during processing, lead to significant variations in their mechanical properties. Overall, PAEK polymers demonstrate excellent shape retention under oxidative and thermal conditions, making them widely recognized HPPs for high‐temperature applications [32, 33]. Table 2 summarizes various material properties of these PAEK polymers.

**TABLE 2:** Comparative intrinsic properties of high‐performance PAEK polymers with different ether‐to‐ketone ratios. Reproduced (Adapted) with permission from [34]. Copyright 2022, John Wiley and Sons.

PAEK Polymers	PEEK (Victrex 150 PF)	PEEK (Victrex 450 PF)	PEKK (Kepstan 6002 T/I = 60/40)	PEKK (Kepstan 7002 T/I = 70/30)	PEKK (Kepstan 8002 T/I = 80/20)	PEK (EOS HP3)
Intrinsic properties						
Ether‐to‐ketone ratio	2:1	2:1	1:2	1:2	1:2	1:1
Processing temperature [°C]	313.4 –333.7	300.2 – 320.5	—	—	—	338.3 –354.2
T_g_ [°C]	∼143	∼143	160	162	165	164
T_m_ [°C]	345	339	300‐305	331	360	372
Crystallinity [%]	46.7 ± 5.2	47.3 ± 5.3	0.5–28.4	3.2–28.1	7.1–34	48.7 ± 5.5
Molecular weight [g mol^−1^]	23,000	32,000	—	—	—	23,000

PIs are also widely recognized HPPs, which have gained attention for their excellent material properties and ease of material processability from their various precursors. Kapton is one of the most renowned PIs, which is commercially graded for multifunctional applications [35, 36]. Like other HPPs, PIs consist of a rigid aromatic chain with a strong imide (‐CO‐N‐CO‐) functional group. Based on their distinct molecular structures, PIs can be formulated as either thermosetting or thermoplastic polymers, resulting in a wide range of industrial applications. In addition, PIs can be categorized into aromatic, semi‐aromatic, and aliphatic polymers, each with distinctive characteristics, making them tailorable to achieve the desired material properties [35, 37, 38, 39]. Together with a relatively low coefficient of thermal expansion (CTE) and high temperature stability (∼473°C–487°C) [40], their aromatic backbone provides remarkable toughness, dielectric performance, and chemical resistance [36]. Despite their substantial advantages, these polymers present significant processing challenges, including poor solubility in most organic solvents and limited flowability, which restricts their use in achieving the desired material properties for demanding applications. To address these issues, PIs are often processed from their precursor form. Poly (amic acid) (PAA) is the most widely used PI precursor, which can be imidized at high temperature (∼300°C) into the desired PI structure for specific high‐temperature applications [41, 42].

PTFE, commercially known as Teflon, is a tetrafluoropolymer that exhibits excellent material properties, including high molecular weight, strong chemical inertness, high temperature stability, and high thermal degradation onset temperature (>400°C) even under exposure to pure oxygen, due to its robust C‐F bonds [24]. Because of its strong C‐F bonds and excellent chemical resistance, PTFE exhibits high resistance to nearly all solvents, bases, and acids. In addition, PTFE exhibits strong hydrophobicity (contact angle ~120°) due to its nonpolar molecular structure and low surface energy. Collectively, these unique properties make PTFE invaluable in various demanding applications, particularly for high‐temperature environments [24, 43, 44, 45].

Taken together, these chemistries provide the thermal and chemical stability required for extreme service, but their rigid backbones, high transition temperatures, restricted chain mobility, and, where applicable, crystallization kinetics also create the rheological and thermal constraints that govern AM processability. The following section examines how FFF, SLS, and DIW accommodate these material‐specific constraints.

## 3 Additive Manufacturing of High‐Performance Polymers (HPPs)

Additive Manufacturing (AM) of HPPs has predominantly relied on fused filament fabrication (FFF), selective laser sintering (SLS), and direct ink writing (DIW) for fabricating structures from these materials. In recent years, researchers have used these AM techniques with HPPs in demanding applications, such as aerospace, automotive, marine, and energy [46, 47, 48, 49, 50, 51]. However, each of these AM techniques faces several challenges for printing HPPs, including high melting temperatures, high melt viscosities, layer delamination, warping, and dimensional shrinkage (Table 3). Due to these challenges, printed HPP structures often exhibit poor print quality, inconsistent performance, limited reproducibility, and inadequate mechanical properties. By optimizing material properties and technical parameters, several studies have demonstrated the AM of HPPs, particularly using PEI, PEEK, PEKK, PEK, PTFE, and PI [46, 47, 48, 49, 50, 51, 52, 53, 54, 55, 56, 57]. This section discusses recent advancements, critical challenges, and remaining limitations of these AM techniques for representative HPPs.

**TABLE 3:** Comparison of additive manufacturing techniques used to process high‐performance polymers.

AM technique	Advantages	Disadvantages	Applications
Fused filament fabrication (FFF)	Lower equipment and material costs compared to SLS. Many HPP filaments that meet industrial design and safety standards are commercially available, such as ULTEM 9085 and Antero 800NA. Tunability of crystallization kinetics through controlling extrusion, chamber, and build plate temperature, print speed, flow rate, raster angle, print trajectory, etc. [47, 48, 49, 51, 54, 58, 59]. Various functional additives and fillers can be integrated into HPP filaments to facilitate multifunctional HPP structures [60, 61].	Requires specialized high‐temperature nozzles and heated chambers (>350°C for PEI, >400°C for PEEK). Large thermal gradients during deposition cause rapid solidification of deposited HPPs, leading to poor interlayer adhesion, warping, dimensional instability, volumetric shrinkage, residual stress accumulation, and entrapped gas and voids. High melt viscosity of HPPs restricts smooth flow through extrusion nozzles, leading to interlayer and intralayer voids and incomplete layer coalescence. The layer‐by‐layer strand deposition of FFF‐printed parts frequently results in irregular and jagged surface finishes, which require intensive post‐chemical surface treatments [62] or in situ high‐power laser‐assisted thermal heating systems [63]. Hygroscopic nature of HPP filaments can degrade filament quality, significantly affecting interlayer bonding and print fidelity. Therefore, FFF printing of HPPs strictly requires pre‐processing, including environmental management and filament drying throughout the storage and printing process [64].	Flight‐certified parts (ducting systems, brackets, avionics enclosures) Intake manifolds Anti‐ballistic shields High‐temperature seals and housings in marine environments. Radiation shielding in nuclear facilities. Lightweight and on‐demand structural components by Stratasys [65], NASA [66], Royal Netherlands Navy's Expertise Center Additive Manufacturing (ECAM) [23], ROBOZE [67], AGH University of Science and Technology [68], and Nanovia [69].
Selective laser sintering (SLS)	No support structures are required, as unfused powder acts as a self‐supporting medium. Powder reusability makes it potentially economical for large‐scale production of 3D‐printed HPPs. Ability to customize meso‐to‐microstructural properties by controlling powder morphology, laser parameters, and bed temperature enables good control over the crystallinity of SLS‐printed HPPs [70]. SLS‐printed HPPs have near‐injection‐molded strength due to superior interlayer fusion resulting from more uniform temperature gradients and efficient particle amalgamation through powder fusion [71]. The mechanical properties of SLS‐printed HPP structures are nearly isotropic when fully sintered.	A very high‐energy laser is required. Excessive energy density can thermally degrade HPPs and sometimes cause charring [72, 73]. Limited commercial availability, as only a few HPP powders have been optimized for SLS. Often requires post‐processing annealing for dimensional stability. Commercially available powder particles of HPPs often exhibit unsuitable properties for SLS, such as a broad molecular weight distribution and irregular particle shapes, which require additional powder processing techniques, including cryogenic grinding and milling, to achieve a finer particle size distribution [34]. Inappropriate laser parameters often cause partial sintering of HPPs, leading to microstructural defects.	SLS‐printed HPPs often exhibit superior performance to their conventionally‐processed counterpart as well as smooth print surfaces, making them suitable for automotive, aerospace, semiconductor, and other high‐temperature applications [74]. Engine manifolds, brackets Flame‐retardant PEI components. PPS composites for offshore structures Superhydrophobic PTFE architectures, radiation shielding (PEEK‐tungsten composites).
Direct ink writing (DIW)	Room‐temperature printing avoids thermal degradation and internal stress accumulation and eliminates the necessity of thermal management systems for the extrusion nozzle, print bed, and build chamber. Enables multiscale (nano‐to‐macro) fabrication of HPPs using solution‐based inks rather than high‐energy heating or melt processing [75]. It offers the flexibility of incorporating various fillers and additives into the precursor inks of HPPs to improve the printability and multifunctionality of HPP structures [76]. UV‐assisted DIW techniques for HPPs promote in situ polymerization of the printed HPP precursor inks, providing highly stable crosslinked structures with improved interlayer adhesion [75].	DIW‐printed HPPs require post‐curing at high temperatures, which often leads to crack formation and large porosity. Ink formulation is complex, requiring rheology modifiers and solvents for printability. Up to 45% dimensional shrinkage during solvent removal and curing. Lower mechanical strength of printed HPP parts compared to FFF/SLS unless reinforced.	High‐temperature and flame‐retardant HPP structures using DIW exhibit suitability for multifunctional applications, including thermal coating, aerospace insulation, and casings for electronic devices.

### 3.1 Fused Filament Fabrication (FFF) Process

In recent studies, several researchers have successfully 3D‐printed HPPs, such as PEI, PEEK, and PEKK, using the FFF process. In FFF, the filaments first undergo extrusion upon melting at high temperatures, followed by layer‐by‐layer deposition to construct intricate 3D architected structures. Figure 2a illustrates the working principle of the FFF process. However, FFF of HPPs faces several challenges, such as poor interlayer adhesion, warpage, volume shrinkage, layer delamination, improper material fusion, surface cracks, void formation, and accumulation of residual stress due to repeated heating and cooling. These challenges primarily stem from the inherent properties of HPPs, such as their high melting point, inadequate flow behavior, and poor surface adhesion [34, 77, 78, 79, 80]. The specific material properties, such as T_g_, T_m_, ductility, crystallinity, viscosity, etc., required to mitigate these challenges must be thoroughly studied to facilitate the manufacturing of high‐quality architected HPP structures.

**FIGURE 2:**
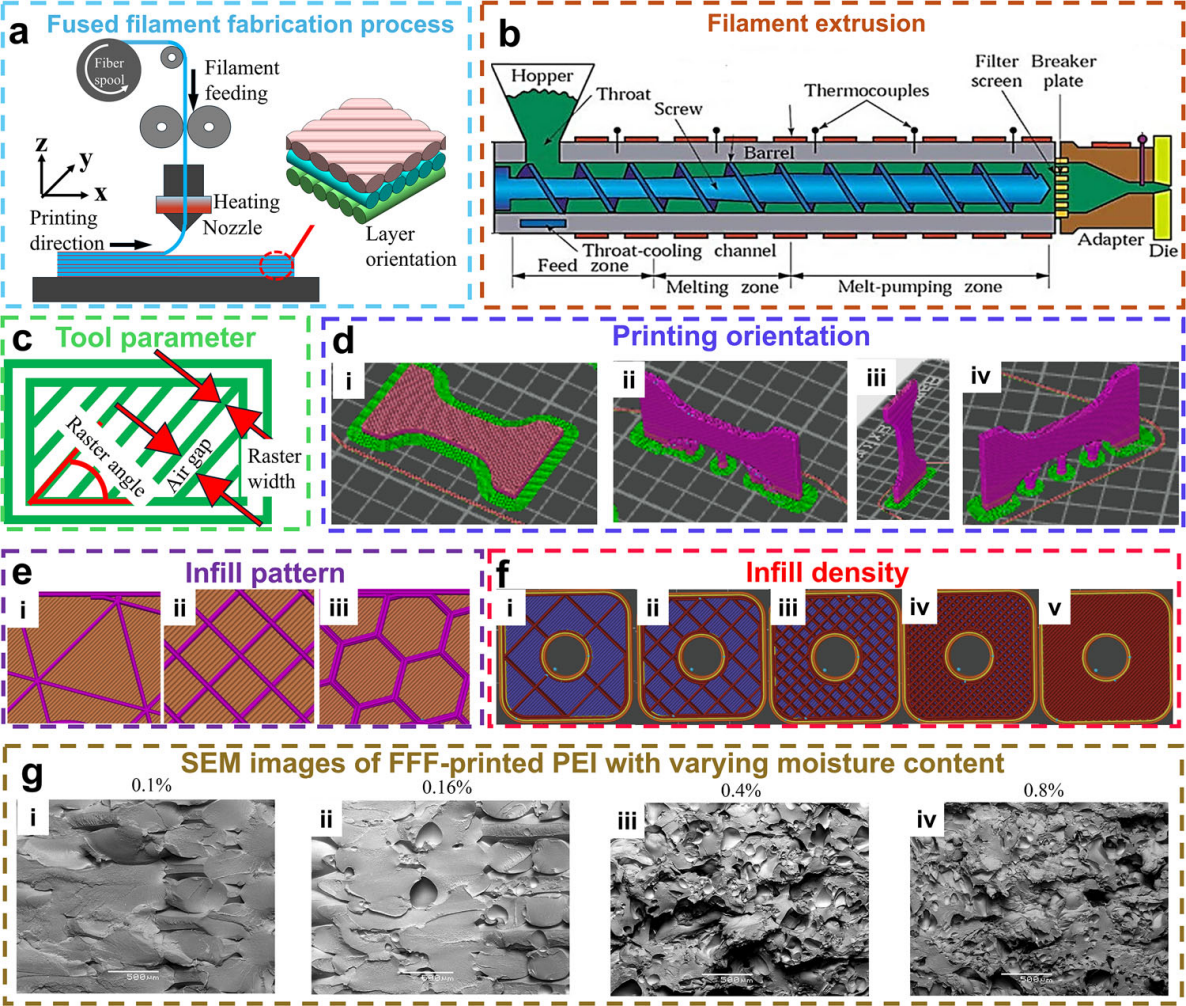
Fused filament fabrication (FFF) process and key processing parameters. (a) Schematic of the FFF process illustrating the filament extrusion through the heated nozzle and layer‐by‐layer deposition. (b) Illustration of the filament fabrication process through an extrusion mechanism. The schematic demonstrates the basic working principle of extruder machines, showing how pellets are transformed into filaments. Reproduced (Adapted) from [81] under the terms of the Creative Commons Attribution 4.0 International License (CC BY 4.0). (c) Key FFF parameters that affect the print quality and mechanical performance of the printed parts, including raster angle, raster width, and air gap. (d) Rendering of printing orientations: (i) flat, (ii) on‐edge horizontal, (iii) on‐edge upright, and (iv) on‐edge inclined. (e) Infill patterns: (i) triangular, (ii) rectilinear, and (iii) honeycomb. (f) Rendering of infill densities for rectilinear infill pattern: (i) 5%, (ii) 10%, (iii) 30%, (iv) 60%, and (v) 80%.  (g) SEM images of FFF‐printed PEI with varying moisture content. Reproduced (Adapted) with permission from [64]. Copyright 2018, Elsevier.

#### 3.1.1 Filament Fabrication Process for FFF of HPPs

Optimization of FFF of any material begins with the filament fabrication process. Filaments used in the FFF method are typically developed from representative HPPs through a filament extrusion process, where an external pressure pushes the polymer at high temperatures through a die to form the filament. The filament quality and precise diameter depend on several factors, such as applied pressure, die temperature, roller puller speed, spindle speed, and intake temperature. Throughout each stage of feed, transition, and metering, granulated polymers undergo softening, plasticization, and melting (Figure 2b). Each of these stages influences the properties of the filaments and, consequently, the final printed parts.

#### 3.1.2 FFF of Polyetherimide (PEI)

To facilitate the AM of architected PEI structures via FFF, the first step is transforming PEI into FFF‐ready filaments. However, converting an HPP such as PEI into filaments presents multiple challenges. Unlike common FFF polymers such as PLA or ABS, PEI (ULTEM 1010) has a high glass transition temperature (220°C–230°C) and requires elevated extrusion temperatures (360°C–380°C) [47]. Early attempts frequently faced challenges such as inconsistent filament quality, brittleness, and susceptibility to moisture absorption. These contributed to nozzle clogging and uneven extrusion during FFF, along with a decline in the mechanical properties of the printed structures. To address these issues, researchers optimized melt rheology and extrusion processes to enhance filament uniformity. Pre‐drying protocols were established to minimize moisture uptake, while spool packaging under controlled conditions reduced hydrolytic degradation during storage. Despite these advances, challenges remain to be addressed. PEI filaments continue to be more expensive than commodity thermoplastics, require specialized high‐temperature nozzles and build chambers, and remain moisture‐sensitive. 3D printing PEI via FFF involves adjusting multiple parameters, including extrusion temperature, flow rate, raster angle, contour width, air gap, and cooling rate. Each of these substantially affects the print quality and mechanical performance of printed parts.

Table 4 presents the optimal values of these specific parameters based on the mechanical and rheological properties. For instance, Ouassil et al. found that printing speed substantially influences the tensile characteristics of FFF‐printed parts, especially when printed in the vertical (Z) direction [47]. On the other hand, the effect of extrusion temperature was minimal. Through optimization of printing speed (∼30 mm/s) and nozzle temperature (∼360°C) with a post‐heat treatment at 230°C for 24 h, they achieved the highest tensile strength of 102 MPa and stiffness of 3425.5 MPa. In another work, Gebisa et al. assessed the effect of raster angle, raster width, contour number, air gap, and contour width on the FFF printing of PEI (ULTEM 9085) (Figure 2c–f) [48]. The most important factor influencing print quality and tensile strength was found to be the raster angle. For example, compared to 0° raster angles, 90° raster angles were more susceptible to changes in the air gap. The fine‐tuning of raster angle played a crucial role in maximizing print quality, minimizing air gaps, and enhancing mechanical properties. They found significantly increased tensile properties of the printed parts by reducing the raster angle from 90° (30 MPa) to 0° (87 MPa). This could be attributed to the decrease in internal voids through the overlapping of the adjacent filaments caused by reduced air gaps at lower raster angles [82]. A minor but noticeable effect of increasing the contour width and number on the tensile properties was also observed. On the other hand, extrusion temperature had a quantifiable effect; the ideal value for optimizing both impact and tensile strength was found to be 370°C [82]. Yilmaz et al. found a similar effect of raster angle on FFF‐printed PEI (ULTEM 1010) components by varying layer orientations [49]. They achieved a maximum tensile strength of 71.0 MPa for samples printed at 0° raster angle and ∼36 MPa for those printed at 90°. Typically, multi‐oriented components perform better than single‐oriented ones, especially in terms of strain‐to‐failure. It is noteworthy that the 30°/−60° raster design shows the maximum tensile strength of 63.4 MPa. Furthermore, compared to single‐orientation, multi‐orientation shows a significantly greater failure strain, leading to improved toughness, which is essential for applications susceptible to fatigue or impacts.

**TABLE 4:** Process parameters for fused filament fabrication (FFF) of PEI.

Parameter	Grade	Optimal value(s)	Effects/Benefits	References
Nozzle temperature	ULTEM 1010	360°C	Maximizes tensile and impact strength.	[47]
	ULTEM 1000	370°C	Optimal tensile and impact strength.	[82]
Printing speed	ULTEM 1010	30 mm/s	Maximizes tensile and impact strength.	[47]
Raster angle	ULTEM 1010	0°	Maximizes tensile strength and storage modulus.	[49]
Raster angle	ULTEM 1010	30°/−60°	Highest strength for multi‐oriented samples.	[49]
Raster angle	ULTEM 9085	0°	Increases tensile strength from 30 MPa to 87 MPa.	[48]
Air gap	ULTEM 9085	Low	Increases tensile strength.	[48]
Layer offset	ULTEM 9085	0.25 mm horizontally	Enhances wear resistance, decreases mass loss.	[58]
Printing direction	ULTEM 9085	Z orientation with a 0.25 mm offset in alternate layers	Up to 127.43% more wear resistance.	[58]
Moisture content	ULTEM 9085	Below 0.16%	Prevents significant mechanical performance reduction.	[64]
Heat treatment	ULTEM 1010	Annealing at 230°C	Reduces the internal porosity, improving interlayer bonding and enhancing properties.	[47]

The layer‐by‐layer strand deposition of FFF‐printed parts frequently results in irregular and jagged surface finishes. To overcome this, various post‐print surface treatments can be performed. Fischer and Schöppner investigated the effect of chemical surface treatment on FFF‐printed PEI (ULTEM 9085) parts [62]. They found that the surface roughness R_z_ can be reduced from 135 to 34 µm through chloroform vapor treatment. Although the tensile strengths in the X‐ and Y‐directions remain unaffected by chemical treatment, the tensile strength in the Z build direction increases by ∼16% after treatment. However, they found a negligible impact of chemical smoothing on the fatigue performance of the printed PEI parts. Other attempts have also been made to improve the tensile strength of PEI (ULTEM 1010) parts in the build direction. One such method involves the introduction of a laser‐assisted thermal heating system, which improves interlayer bonding [83]. These results highlight the importance of post‐processing methods and parameter adjustments in producing high‐quality, structurally sound PEI components using FFF.

In‐layer deposition strategies and sample building orientations can also be optimized to enhance the mechanical properties of FFF‐printed PEI structures. Based on geometric configurations, Mitaľ et al. developed a novel trajectory adjustment that included an offset of 0.25 mm along the horizontal direction during the layer deposition [58]. The wear resistance of this unique stacking sequence was assessed in both X‐ and Z‐directions. Compared with traditional vertical stacking, the improved stacking method reduced weight loss due to wear by 65.44%. In particular, the FFF‐printed components in the X orientation with a 0.25 mm offset in alternate layers were 22.92% less durable than those fabricated using perpendicular layering, whereas the Z‐oriented components with a 0.25 mm offset exhibited up to a 127.43% improvement in wear resistance compared with perpendicular layering. These findings emphasize the significance of the stacking technique and printing direction in optimizing wear performance, particularly for tribologically demanding applications. Overall, to maximize wear resistance in FFF‐printed PEI components, this study suggests employing print configurations along the Z‐axis with offsets in the horizontal direction [58].

Besides geometric configurations, environmental factors such as moisture absorption also significantly influence the print quality and mechanical performance of FFF‐printed PEI structures (Figure 2g). Zaldivar et al. found that strain‐to‐failure can be reduced by 41% with as little as 0.16% moisture absorption [64]. There was a linear decrease in failure strain with increasing moisture content. However, at low moisture concentrations (<0.1%), they found an increase in modulus and strength. This was due to improved coupling between printed filaments, resulting from increased flow during extrusion [64]. They developed diffusion models to calculate the time required for complete drying before printing. To preserve the mechanical integrity of FFF‐printed structures, these results highlight the crucial need for pre‐processing, including environmental management and filament drying, throughout storage and printing.

To summarize, these studies identify raster orientation, layer trajectory, and filament moisture as the dominant controls that govern mechanical strength and wear resistance of FFF‐printed PEI structures. Raster orientation and layer trajectory influence interlayer bonding and load transfer, whereas moisture uptake can promote extrusion instability and defect formation because of the hygroscopic nature of PEI. Chemical smoothing and localized thermal assistance can improve surface quality and interlayer bonding; however, reliable moisture management remains essential for reproducible printing.

#### 3.1.3 FFF of Poly(Ether Ether Ketone) (PEEK)

Additive manufacturing of PEEK via FFF presents additional challenges due to its even higher melting point, unfavorable melt rheology, rapid crystallization dynamics, and high susceptibility to temperature gradients. Despite these challenges, some optimized material formulations and process parameters have facilitated the fabrication of complex architected PEEK structures via FFF (Table 5). Multiple studies have focused on improving the print quality and mechanical properties of FFF‐printed PEEK structures by tuning nozzle temperature, chamber conditions, printing orientations, and printing speed.

**TABLE 5:** Recommended values of FFF processing parameters for PEEK from different filament manufacturers. Adapted from reference [4] under the terms and conditions of Creative Commons Attribution (CC BY 4.0) license.

Brand name	Nozzle temperature (°C)	Bed temperature (°C)	Printing speed (mm/s)	Chamber temperature (°C)	Bed preparation
ThermaX^TM^	375–410	130–145	10–50 for 0.2 mm thickness	70–140	ULTEM Tape
3D4MAKERS	360‐400	120	15–30	70–150	PEI sheet
Polyfluor^TM^	335–390	120	—	—	—
KetaSpire	390–420	>200	—	—	—
LUVOCOM	370–420	>120	—	—	—

To address thermal management concerns, a heated print chamber is necessary for the 3D printing of PEEK, with advanced cooling systems that incorporate mixed convection heat transfer. Park et al. demonstrated that maintaining an elevated chamber temperature, up to 160°C depending on the printing orientation, reduced thermal gradients and associated thermal stresses, thereby suppressing interlayer delamination and warpage in FFF‐printed PEEK [51]. This slow crystallization process enabled the development of superior mechanical properties. Wang et al. also conducted a similar study on carbon fiber (CF)‐ and glass fiber (GF)‐reinforced PEEK filaments to optimize different printing parameters, particularly the extrusion temperature, build temperature, print speed, and layer thickness. Keeping all other factors unchanged, they found that increasing the nozzle temperature (from 400°C to 440°C), increasing the build plate temperature (from 240°C to 280°C), decreasing the layer thickness (from 0.3 mm to 0.1 mm), and reducing the printing speed (from 25 mm/s to 5 mm/s) improved the mechanical properties and surface textures of FFF‐printed parts [84]. Internal defects were also reduced due to better binding strength between layers and infill filaments. Furthermore, Yang et al. showed that with an increase in ambient temperature, crystallinity increases significantly from 17% at 25°C to 31% at 200°C, and mechanical properties improve accordingly [54]. Additionally, for post‐print treatment, they found that furnace cooling and annealing yield higher crystallinity compared to tempering or quenching methods.

Regarding printing orientation, Park et al. demonstrated that printing along the longitudinal direction shows the least tendency to warpage compared to the transverse direction [51]. Furthermore, Arif et al. conducted an extensive study on the impact of build orientation (Figure 3a) and thermal gradient (Figure 3b) effects on the print quality and mechanical properties of FFF‐printed PEEK [59]. They showed that the horizontal (H‐0) printed samples exhibit higher mechanical properties in comparison to H‐90 (Figure 3c). This is because the layer orientations of H‐0 samples are directly aligned with the loading direction. However, due to longer deposition paths and the rapid solidification of the deposited filaments, the H‐0 samples exhibit weaker interlayer adhesion compared to the H‐90 samples. During the build process, the H‐90 configurations exhibit a lower thermal gradient compared to H‐0 and V‐90, which slows the solidification process and thereby improves bead bonding. Therefore, the H‐90 configuration exhibits comparable mechanical properties, despite maintaining a constant print speed and varying build rates. These findings suggest that a balance between the build configuration and build rates is crucial for optimizing the print quality and mechanical properties.

**FIGURE 3:**
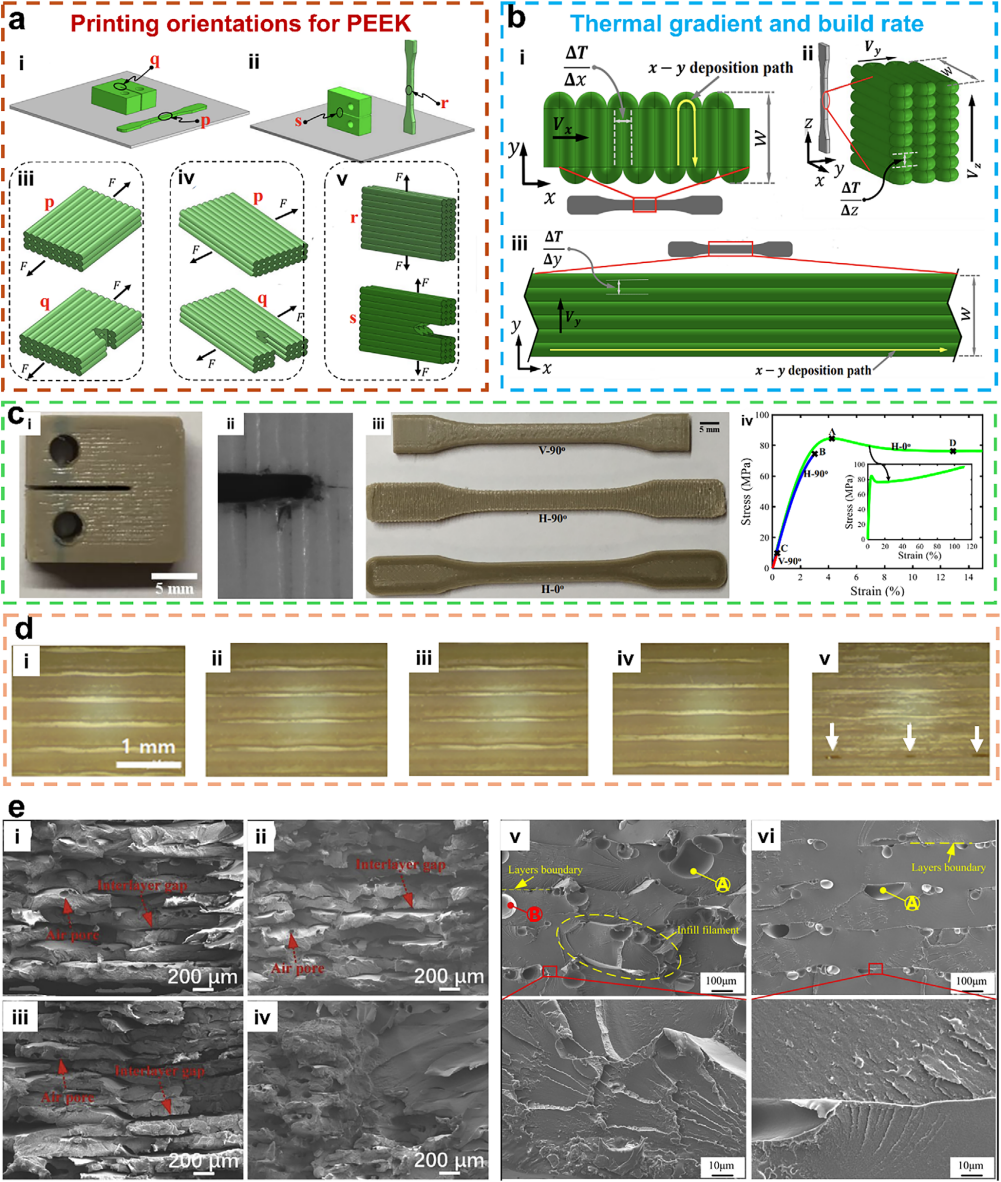
FFF processing of PEEK: (a) rendering of FFF configurations for mechanical compact tension and tensile specimens, fabricated in two different orientations: (i) horizontal and (ii) vertical. For horizontally fabricated specimens, the deposition patterns included (iii) 0° bead orientation and (iv) 90° bead orientation, while vertically fabricated specimens had (v) 90° bead orientation. Sub‐figures p and q in (iii) and (iv) provide a magnified view of regions p and q in (i), respectively. Similarly, sub‐figures r and s in (v) show the enlarged zones of regions r and s in (ii). The configuration of flexural specimens closely resembles that of the tensile specimens. (b) A schematic representation of the thermal gradient and build rate during the FFF‐PEEK process for (i) H‐90°, (ii) V‐90°, and (iii) H‐0° samples. The build rate for H‐90° samples is represented as $v_{x}=\frac{\Delta x}{\Delta t}$ in the *x* direction, for H‐0° and V‐90° samples as $v_{y}=\frac{\Delta y}{\Delta t}$ in the *y* direction, and for H‐0°, H‐90°, and V‐90° samples as $v_{z}=\frac{\Delta z}{\Delta t}$ in the *z* direction. (c) FFF‐printed PEEK samples: (i) printed compact tension specimens, (ii) magnified view of the crack tip of the compact tension specimen, (iii) printed specimens with different bead orientations for tensile test, and iv) corresponding stress‐strain curve. Reproduced (Adapted) with permission [59]. Copyright 2018, Elsevier. (d) Optical images of FFF‐printed PEEK at different nozzle temperatures: (i) 390°C, (ii) 400°C, (iii) 410°C, (iv) 420°C, and (v) 430°C. Reproduced (Adapted) with permission [51]. Copyright 2021, Elsevier. (e) SEM images of fracture surfaces under tensile tests of the 3D‐printed PEEK specimens at different nozzle temperatures: (i) 370°C, (ii) 380°C, (iii) 390°C, (iv) 420°C, and representative SEM images at (v) 380°C and (vi) 440°C. Point A indicates the voids between the successive printed layers, while point B indicates the voids between the infill extruded filaments. Reproduced (Adapted) with permission [85]. Copyright 2019, Elsevier.

Another dominant factor is the nozzle temperature, which determines the microstructure and print quality. Park et al. showed that the stable printing of PEEK is possible at a minimum nozzle temperature of 390°C (Figure 3d(i‐iv)) [51]. However, significant thermal degradation and voids can occur beyond the nozzle temperature (∼430°C) (Figure 3d(v)). These voids can impede the smooth extrusion of filament through the nozzle and adversely affect printing quality. After setting these optimized parameters, they achieved 3D‐printed samples with a maximum tensile strength of 87.5 MPa. This suggests that ideal thermal conditions promote enhanced material consolidation and interlayer diffusion. Wang et al. showed that a positive correlation exists between nozzle temperature and the density of 3D‐printed PEEK, where elevated temperatures lead to an increase in density [86]. Ding et al. found that the comparative micrographs of PEEK samples printed at 380°C and 440°C reveal a significant reduction in both the number and size of voids at elevated temperatures (Figure 3e) [85]. They also found that the optimal extrusion temperature for mechanical performance is between 420 and 440°C. Yang et al. verified that higher extrusion temperatures promote the crystallization kinetics, which enhances tensile strength and stiffness [54]. These findings suggest that optimized nozzle temperatures can effectively minimize porosity and internal defects, leading to improved density and potential enhancements in the mechanical properties of FFF‐printed PEEK components.

Collectively, these studies show that FFF‐printed PEEK is governed by a narrow balance between sufficient thermal input for melt consolidation and excessive exposure that promotes degradation or undesirable crystallization. Chamber temperature, nozzle temperature, cooling history, and build orientation therefore act as the principal controls on porosity, interlayer bonding, and mechanical anisotropy.

#### 3.1.4 FFF of Poly(Etherketoneketone) (PEKK)

PEKK copolymers demonstrate tailored crystallization and thermal behavior based on their chemical structure, specifically the percentage of terephthaloyl (T) to isophthaloyl (I) components. In general, commercial grades fall between 60/40 and 80/20 (T/I), where a higher T content raises the melting point much more strongly than the glass transition temperature [55]. Due to its more amorphous region and reduced shrinkage and warpage during cooling, PEKK with a lower T/I is better suited for the FFF technique. Nevertheless, issues such as inadequate interlayer bonding, excessive melt viscosity, and microstructural gaps continue to impair the structural integrity and mechanical properties of FFF‐printed PEKK components. Researchers investigated both process‐ and formulation‐based approaches to overcome these constraints. In a pivotal work, carbon fiber (CF)‐reinforced PEKK filaments were successfully FFF printed using a specially designed desktop printer, highlighting the need to precisely adjust processing temperatures to achieve ideal flow characteristics and robust interlayer bonding [87]. In addition to controlling layer‐wise welding, rheological properties impact crystallization kinetics and molecular orientation, which in turn affect the ultimate mechanical properties. Complementary research has further described the viscoelastic dynamics of molten PEKK during extrusion processes [88]. To address the challenges posed by high viscosity and improve print quality for consistent results at larger scales, researchers made adjustments to factors including extrusion temperature, filler selection, and shear rate [60].

Rashed et al. investigated the impact of several FFF parameters through Taguchi Design of Experiments (DOE) statistics, utilizing infill patterns, build orientation, raster angle, and number of contours [55, 77]. With the help of this statistical tool, the effects of each of these parameters on the tensile properties of PEKK were carefully investigated. The results revealed that build orientation is the dominant factor governing mechanical properties and strain at failure, which aligns with the findings of Yilmaz et al. [49]. Additionally, the number of contours was found to have a significant impact on these properties. Notably, samples printed in a horizontal orientation exhibited a higher Young's modulus compared to those printed vertically. Furthermore, variations in cooling rates influence the microstructure, material flow rate, and degree of crystallinity. Their findings also indicate that while linear infill patterns enhance mechanical properties, gyroid infill patterns exhibit superior material stiffness, likely due to improved interlayer adhesion. Micro‐computed tomography (Micro‐CT) analysis revealed substantial differences in porosity among the specimens (0.33%–2.89%), with vertically printed samples exhibiting greater, more heterogeneous porosity. This increased porosity compromised mechanical properties, and the results suggested that optimizing printing parameters could minimize it. Overall, they have found that the number of contours plays a major role in determining the mechanical properties of the printed parts. On the other hand, the raster angle was found to influence ductility and toughness significantly. Table 6 summarizes the process parameters and corresponding optimal values for AM of PEEK and PEKK polymers using the FFF technique.

**TABLE 6:** Optimal FFF processing parameters and their effects on the printed objects of PEEK and PEKK.

Parameter	Optimal value	Polymer	Effects/Benefits	References
Raster angle	0° (horizontal)	PEEK	Young's modulus: 3.80 GPa, Tensile strength: 82.58 MPa, Flexural strength: 142.0 MPa	[59]
Layer thickness	0.3 mm	PEEK	Best strength and surface quality	[89]
Nozzle temperature	390–420°C	PEEK	Maximizes mechanical properties	[85]
Nozzle temperature	380–440°C	PEEK	Best results with optimized feeding speed	[86]
Printing speed	15 mm/s	PEEK	Optimal for surface roughness	[90]
Post‐processing	Annealing	PEEK	Enhances mechanical properties	[54]
Build orientation	Horizontal	PEEK	Better mechanical properties than vertical	[59]
Printing temperature	375–390°C	PEKK	Strong interfacial bonds and reduced viscosity	[60]
Build orientation	Horizontal	PEKK	Higher Young's modulus due to cooling rates	[55]
Infill pattern	Gyroid	PEKK	High modulus through improved interlayer adhesion.	[55]
Number of contours	High	PEKK	Enhanced tensile modulus	[55]
Raster angle	± 45°	PEKK	Increased ductility	[55]

To sum up, PEKK offers a comparatively adjustable crystallization response, particularly through control of the terephthaloyl/isophthaloyl ratio; however, high melt viscosity and orientation‐dependent porosity continue to limit printing consistency and quality. Build orientation, contour number, infill strategy, and cooling history should therefore be treated as connected variables rather than optimized independently.

#### 3.1.5 Current Challenges and Mitigation Strategies for FFF of HPPs

The major challenges associated with the FFF technique of HPPs include insufficient interlayer adhesion, warping, dimensional inaccuracy, poor surface finish, defect formation, and porosity. These limitations arise from the combined influence of material properties and processing conditions. For instance, rapid cooling caused by the large temperature gradient between the heated nozzle and the previously deposited layers limits polymer‐chain diffusion and, in semicrystalline polymers, restricts crystal growth at the layer interfaces. Consequently, incomplete bonding may develop between adjacent layers, leading to weak interlayer adhesion and anisotropic mechanical properties in the printed structures. The moisture content of hygroscopic filaments can further affect melt flow, extrusion stability, and adhesion between successive layers. However, the use of a heated build platform, a temperature‐controlled chamber, and a high‐temperature nozzle can reduce thermal gradients and slow the cooling rate, thereby extending the time available for polymer‐chain diffusion and improving interlayer bonding. Post‐thermal treatments, including annealing and autoclave heating, can also promote interlayer consolidation, crystal growth, and residual‐stress relaxation [4, 51, 54, 64, 85, 86].

Warping is primarily caused by volumetric shrinkage during rapid solidification, poor adhesion between the initially deposited layers and the print bed, and nonuniform temperature gradients throughout the printed structure. These effects may be reduced by using a heated build platform, suitable adhesion‐promoting surfaces such as PEI sheets, glue, or tape, design‐based dimensional compensation, and appropriate adjustment of printing parameters, including infill pattern, infill density, and wall thickness. Thermal contraction and volumetric shrinkage during solidification are also major contributors to dimensional inaccuracies in FFF‐printed HPP structures. Therefore, appropriate correction factors for volumetric shrinkage should be incorporated during component design to improve dimensional fidelity.

Void and pore formation in FFF‐printed HPP structures can originate from several sources, including nozzle clogging caused by high melt viscosity, insufficient extrusion temperature, inadequate material flow, moisture‐induced volatilization, incomplete fusion between adjacent extruded filaments, and unsuitable layer thickness. These defects may be reduced by optimizing the material flow rate, tailoring the melt rheology, and selecting appropriate infill patterns, infill densities, layer thicknesses, and shell dimensions. Additionally, post‐processing techniques like annealing, thermal curing, and hot pressing can significantly reduce voids and porosity. Addressing these coupled material‐ and process‐related challenges will be essential for improving the reproducibility and scalability of FFF‐printed HPP structures and expanding the range of polymers that can be reliably processed for load‐bearing and high‐temperature applications.

#### 3.1.6 Critical Synthesis and Research Priorities for FFF of HPPs

Although the studies discussed above have successfully demonstrated FFF of major HPPs, including PEI, PEEK, and PEKK, through optimization of printing parameters and post‐processing conditions, direct comparison among the reported findings remains difficult. The identified processing windows and resulting mechanical properties vary considerably because of differences in polymer grade, molecular weight, filament preparation, moisture content, printer configuration, chamber design, specimen geometry, and testing protocols. In addition, many studies focus primarily on individual processing parameters without considering the combined effects of material chemistry, thermal history, melt‐flow behavior, crystallization kinetics, and residual‐stress development. Consequently, several reported optimum conditions remain specific to a particular material‐printer system and cannot be directly transferred to other HPP grades or FFF platforms.

Thermal management provides a representative example of these inconsistencies. Several studies have demonstrated that an elevated chamber temperature can reduce thermal gradients, delay solidification or crystallization, improve interlayer diffusion, and minimize residual‐stress accumulation and structural warpage. However, other studies indicate that chamber heating alone is insufficient to ensure optimum print quality and mechanical performance unless the post‐thermal treatment is also carefully controlled. Chamber heating primarily influences the thermal history during deposition, whereas post‐print annealing promotes structural relaxation and may alter crystal growth and orientation after fabrication. Therefore, these approaches should not be considered interchangeable. Instead, their individual and combined effects should be evaluated with respect to polymer chemistry, part geometry, cooling history, and the intended balance between dimensional stability and mechanical performance.

Similar limitations exist in the reported effects of nozzle temperature, printing speed, and layer thickness. For instance, an elevated nozzle temperature helps promote polymer diffusion and enhances interlayer bonding. However, the prolonged duration of the melt pool inside the nozzle at excessive melt temperatures may cause polymer oxidation or thermal degradation, hence affecting the overall print quality. Print speed, therefore, can play a crucial role in overcoming this concern. Lower print speed ensures sufficient time for melt pool extrusion, minimizes void content, and improves interlayer adhesion. However, the effectiveness of print speed strongly depends on other crucial factors, such as elevated chamber temperature, polymer rheology, layer thickness, and filament moisture content. Therefore, it is difficult to draw a conclusion on optimizing the mechanical performance and print quality of FFF‐printed HPPs based on a single parametric study.

A further limitation is the absence of standardized protocols for filament conditioning, thermal‐history measurement, defect characterization, and mechanical testing. Future studies should therefore report complete processing histories, adopt consistent drying and testing procedures, and distinguish clearly between interlayer and intralayer defects. Greater emphasis should also be placed on in situ monitoring, numerical modeling, and closed‐loop control of temperature, material flow, and deposition conditions. These developments will be essential for establishing transferable process‐structure‐property relationships and improving the reproducibility and qualification of FFF‐printed HPP components for extreme‐service applications.

### 3.2 Selective Laser Sintering (SLS) Process

Selective laser sintering (SLS) is a type of powder bed fusion (PBF)‐based AM technique, where a high‐intensity heat source, usually a laser, is used to selectively fuse fine powders layer‐by‐layer into 3D objects (Figure 4a). It is one of the widely used AM techniques, particularly for a wide range of thermoplastic polymers. One of the key benefits of SLS is the ability to customize meso‐ to microstructural properties, facilitating the fabrication of architecturally intricate and functional structures. Compared to other AM techniques, the mechanical properties of SLS‐printed structures can nearly resemble those of their injection‐molded equivalents [71]. This results from more uniform temperature gradients and efficient particle amalgamation through powder fusion. SLS provides better interlayer bonding, uniform layer density, and material isotropy [91, 92, 93]. As a result, this technique continues to draw interest in applications that require mechanical integrity, design complexity, and heat resistance, along with the growing availability of various polymer powders. Furthermore, in contrast to the other AM techniques, this one uses the unfused powder as a self‐supporting structure, eliminating the need for additional support materials. This distinct feature increases the reliability of the printing trajectories and streamlines post‐processing. However, the success of this process for high‐quality and functional products depends on several factors. Laser power is one of the most crucial factors that influence the mechanical strength and print quality of the SLS‐produced items. Higher energy densities in SLS increase particle coalescence, thereby improving mechanical properties. However, exceeding a threshold can also degrade the material. Additionally, other critical factors include powder shape, morphology, and flowability, as well as melt‐pool flow dynamics, solidification behavior, and particle coalescence. HPPs that have been successfully printed so far via SLS include PAEK, PTFE, and PEI, each presenting both common and unique challenges.

**FIGURE 4:**
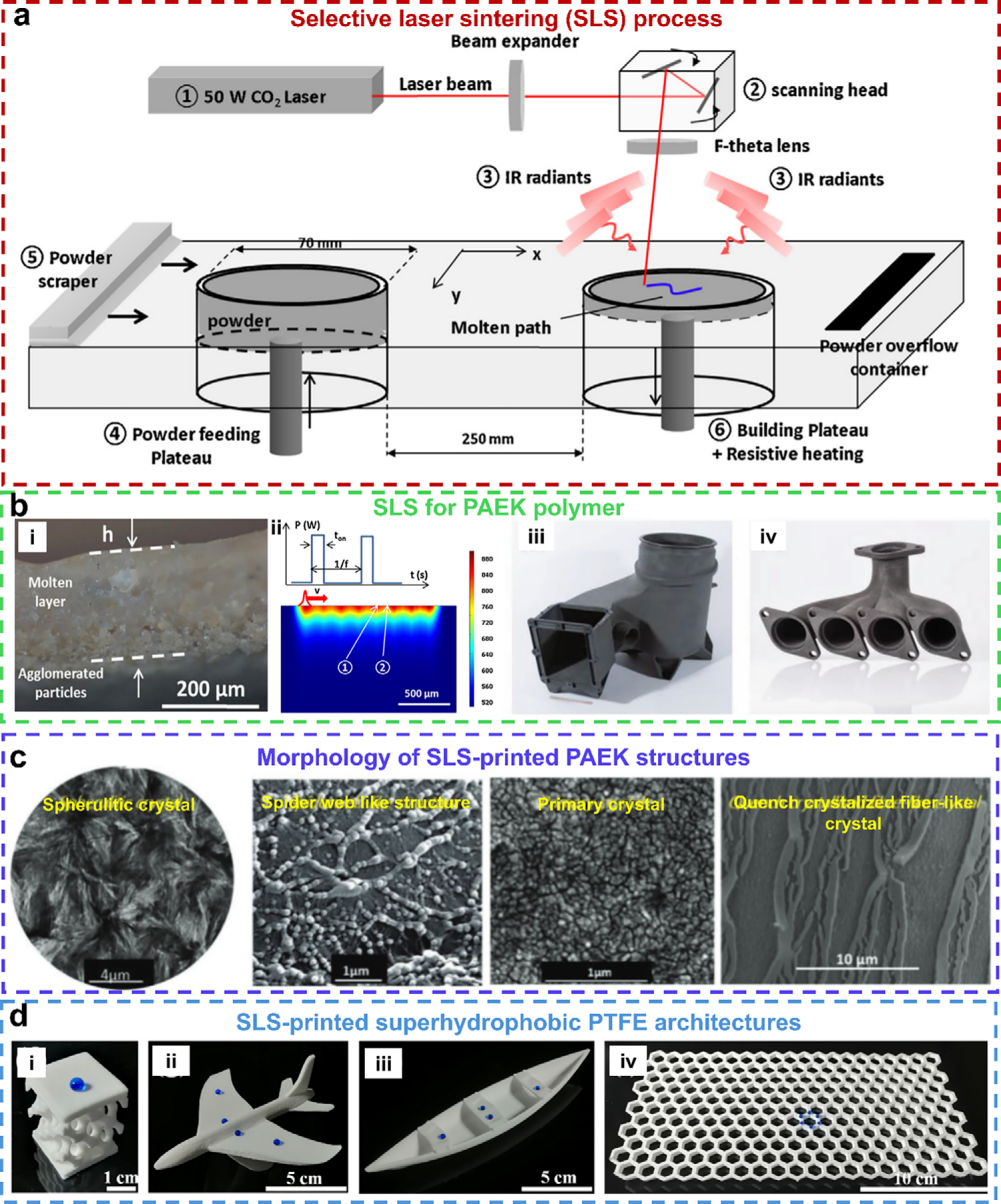
Selective laser sintering (SLS) of high‐performance polymers, including PAEK and PTFE systems. (a) A schematic illustration of the SLS process. Reproduced (Adapted) with permission [57]. Copyright 2015, Elsevier. (b) SLS printing of PAEK polymers: (i) laser sintering of the PEKK powder bed under specific printing conditions to determine the fusion depth (mean laser power, *P_o_* = 3 W; scanning speed, *V* = 0.3 m/s; and initial powder bed temperature, *T_o_* = 600 K), (ii) computational analysis of laser sintering of PEKK powder to investigate the effects of sintering mode (either pulse‐width modulation (PWM) or continuous wave (CW)) on powder fusion, (iii) SLS‐printed component designed by OXFAB ESD for Boeing CST‐100 Starliner, and (iv) an SLS‐printed engine manifold using HT‐23 composite consisting of PEKK polymer with 23% CF reinforcement. b(i‐ii) Reproduced (Adapted) with permission [57]. Copyright 2015, Elsevier, and b(iii‐iv) Reproduced (Adapted) with permission [34]. Copyright 2022, John Wiley and Sons. (c) Crystalline hierarchy of SLS‐printed PEEKs: spherulitic, granular blocks, spherulites, and primary crystals. Reproduced (Adapted) with permission [34]. Copyright 2022, John Wiley and Sons. (d) Printed architectures demonstrating the intrinsic superhydrophobic nature of PTFE: (i) Porous cube, (ii) Aircraft, (iii) Boat, and (iv) Honeycomb matrix (30 cm in length), with the printing parameters of 22 wt% PTFE content and a laser energy density of 0.0625 J/mm^2^. Reproduced (Adapted) with permission [46]. Copyright 2023, Elsevier.

#### 3.2.1 SLS of Poly(Aryl Ether Ketone) (PAEK)

The SLS process has emerged as a large‐scale pioneering technique in AM, particularly for PAEKs such as PEEK and PEKK. However, these commercially available particles often exhibit unsuitable properties for SLS, such as a broad molecular weight distribution and irregular particle shapes [34, 94]. To address these issues, additional processing techniques are essential, including finely meshing through cryogenic grinding and milling. This grinding process enhances the powder characteristics and ensures improved processability, making it suitable for the SLS technique. Various methods are employed to modify the powder characteristics, including thermal conditioning, thermally induced phase separation (TIPS), the addition of fillers and additives, gas‐driven methods, and solvent exchange methods [95, 96, 97, 98, 99, 100, 101]. Among these, thermal conditioning of specific PEEK powders, such as PEEK 450PF and PEEK 150PF, significantly improves particle morphology. This is because it significantly increases particle circularity and roundness, which enhances the flow performance and printability using the SLS technique [101, 102]. On the other hand, the TIPS method modifies PAEK powders by dissolving the matrix in a solvent at elevated temperatures, followed by liquid‐liquid phase separation, droplet nucleation, and precipitation during cooling. This process yields powders with regular spherical shapes and enhanced flowability, making them suitable for the SLS process. Furthermore, the addition of specific additives, such as Aerosil 200 Pharma, significantly improves powder properties, including increased flowability, reduced interparticle forces, and enhanced free flow. Besides these, the incorporation of such additives optimizes powder bed stability, facilitating SLS printability [34, 97].

The interfacial bonding during SLS printing primarily depends on the powder fusion behavior, which in turn depends on the laser power (*P_o_*), scanning speed (*V*), and initial powder bed temperature (*T_o_*). Peyre et al. investigated the effects of these factors on PEKK powder particles based on both experimental and computational studies [57]. They estimated the fusion depth (*h*) by ignoring the unfused agglomerated particles based on optical microscopy (Figure 4b(i)), and they found a linear correlation of *h* with *P_o_/V^0.5^*. This linear correlation gives a fundamental insight into predicting the suitable laser power and sintering speed for complete coalescence of PEKK powder. Furthermore, based on the computational analysis, they found that as long as *V* << *V_max_*, the variation in scanning modes has a negligible effect on the powder fusion by inducing both pulse width modulation (PWM) and continuous wave (CW) modes (Figure 4b(ii)). In another work, Ji et al. prepared PEEK microspheres via a gas‐driven and solvent exchange method, followed by high‐temperature/pressure hydrothermal treatment [103]. This method enables the preparation of PEEK microspheres with super‐smooth surfaces and ensures suitable powder characteristics for successful SLS applications. The degree of crystallinity is another crucial parameter affected by *P_0_*, *V*, and *T_0_*. Wang et al. found a unique hierarchical crystal structure in SLS‐printed PEEK samples, comprising three distinct morphologies within the spherulites: granular crystal blocks, primary crystals, and secondary structures (Figure 4c) [70]. They found that the SLS‐printed PEEK samples exhibit spherulites with a size of 6 µm, while the granular blocks spanning 75–145 nm show a tendency to self‐arrange into spherulitic arrays. This systematic crystallinity improves bulk performance and is necessary for load transmission between the printed layers. Furthermore, they emphasized that the power supply and the temperature evolution of the encompassing unfused powder have a significant impact on the creation and functionality of these crystalline regions. Thus, energy density (ED) and bed temperature can be optimally adjusted to ensure uniform fusion and recrystallization, as they directly impact the degree of crystallinity in the finished product. Overall, microstructural customization via thermal and ED management is essential to utilize the potential of the SLS technique for printing high‐quality, intricate structures of HPPs [70].

Researchers have also investigated PEKK materials, such as Kepstan 6002, to determine the optimal laser parameters for the SLS process. The majority of researchers have used a total ED of 23.5 mJ/mm^2^ per layer of 120 µm for uniform particle fusion, good interlayer adhesion, and good print quality. Besides the PD, precise temperature control, including bed temperature (T_bed_) and building platform temperature (T_bp_), is crucial for improving structural integrity, reducing crack formation, and minimizing layer delamination. Benedetti et al. suggested that T_bed_ and T_bp_ should be 292°C (ideal temperature for PEKK powder coalescence [101]) and 190°C, respectively, to increase the degree of crystallinity with enhanced particle fusion [104]. In order to minimize the danger of thermal damage, they also recommended that a high T_bed_ is necessary to lessen the dependence on high laser PD. On the other hand, Niino et al. utilized the SLS technique to print PEEK polymer at 200°C using a modified laser beam profile for more efficient optical energy use [105]. This method is highly suitable for applications that require high porosity content, such as cell scaffolds. Another study successfully printed a few high‐quality samples with SLS using PEEK and CF/PEEK composites, demonstrating the potential of the SLS technique for high‐temperature and demanding applications (Figure 4b(iii‐iv)) [34].

Regarding the printability of PEK polymer (commercial‐grade PEK HP3) using the SLS technique, Berretta et al. [102] have performed a thorough investigation to understand the characteristics of its particle fusion and coalescence. They found that the faster coalescence from lower‐viscosity PEK produces denser but weaker parts under tensile loading. Although PEK's reduced viscosity due to its lower molecular weight facilitates particle fusion, the polymer is inherently weaker on a molecular level.

Together, these investigations demonstrate how component density, structural integrity, and microstructure are controlled by the interaction of PD, temperature profile, powder rheology, and powder morphology. The optimal parametric values for SLS printing of PAEK polymers are tabulated in Table 7.

**TABLE 7:** Optimal processing parameters for PAEK in SLS technique.

Parameter	Optimal value(s)	Polymer	Effects/Benefits	References
Powder size	Average particle size of 60 µm	PEEK	Enhanced flowability and sintering behavior	[98]
Heat treatment	330–340°C for 2–12 h	PEEK	Reduced angle of repose, improved flowability	[106]
Energy density (ED)	23.5 mJ/mm^2^ per layer	PEKK	Optimal mechanical properties	[104]
Bed temperature (T_bed_)	292°C	PEKK	Successful printing with minimal degradation	[104]
Build platform temperature (T_bp_)	190°C	PEKK	Successful printing with minimal degradation	[104]
Laser parameters	Modified laser beam profile	PEEK	Efficient optical energy use	[105]
Particle size control	Fine and uniform particles via cryogenic grinding	PEEK	Optimal flowability and print quality	[107]
Powder flow enhancements	Aerosil 200 Pharma Additive	PEEK	Improved flowability and anti‐caking properties	[97]
Particle fusion control	Optimized laser energy input	PEEK	Proper particle fusion, improved mechanical properties	[108]
Flowability and sintering quality	Surface treatments/ nanoparticle addition	PEEK	Enhanced flowability and sintering qualities	[97]
Sintering conditions	High temperatures	PEKK	Minimized shrinkage, suitable for high‐temperature applications	[57]
Economic benefits	High degree of powder reuse	PEKK	Consistent rheological properties over multiple builds	[109]

#### 3.2.2 SLS of Polytetrafluoroethylene (PTFE)

Several composite formulations have been developed to address the challenges associated with additive manufacturing of PTFE using the SLS technique. Wu et al. combined PTFE with polypropylene (PP) to form a composite powder that facilitated the AM of PTFE/PP parts using the SLS technique [46]. PTFE was uniformly dispersed within the PP matrix and acted as a catalyst to promote hydrophobicity. On the other hand, PP provided the mechanical framework, contributing to stiffness and printability. The powdered materials were first uniformly combined in a ball mill mixer at a speed of 600 rpm for 15 min, followed by filtration through a 100‐mesh screen to prevent precipitation during the printing process. In this study, a 50 W CO_2_ laser with a scanning speed of 4000 mm/s was utilized to print the PP/PTFE composites using the HUAKE 3D S320 SLS 3D printer. They investigated the effects of different PTFE/PP grains (0–25 wt.%) on hydrophobicity and mechanical strength. Based on mechanical strength and surface wettability, 22 wt.% PTFE was identified as the optimal composition for SLS printing. Thus, they were able to print complex 3D superhydrophobic architectural structures using PTFE/PP grains, as shown in Figure 4d(i‐iv), and the printed structures significantly repelled water droplets. This demonstrates the adaptability of the SLS process in facilitating intricate architected HPP structures.

#### 3.2.3 SLS of Imide‐based Polymers

Limited research has been conducted on the SLS technique to print PEI powder. Although FFF exhibits various drawbacks in achieving good‐quality prints compared to SLS, it has been used in the majority of AM studies on PEI polymer [73]. Due to the high glass transition, high melt viscosity, and rigid amorphous molecular structure of PEI, the successful incorporation of SLS printing remains significantly challenging. Vaganov et al. investigated the viability of SLS using PI powders synthesized by either chemical or thermal imidization [50]. They found that chemically imidized powders demonstrated higher bulk density, narrower particle size distribution, and lower crystallinity in comparison to thermally imidized powders. Consequently, SLS processing of chemically imidized powders produced denser films. This improved mechanical properties, with strength approximately 2.5 times higher and an elastic modulus approximately 2 times higher than that of films derived from thermally imidized powders. They also identified an optimal laser power of 65 W for processing both powders. Additionally, post‐thermal annealing for more than an hour at temperatures exceeding 280°C can enhance the structural integrity through the imidization and crystallization processes of the SLS‐printed PEI parts.

Yifan et al. used PEI powder to impart inherent flame‐retardant characteristics to SLS‐printed parts [73]. In this study, a custom‐built SLS system with a 60 W CO_2_ laser was utilized. The findings indicate that laser power, scan speed, and beam diameter are significant parameters controlling print quality. Lower laser power (10.2 W) resulted in weakly bound particles and extremely fragile printed parts, attributed to inadequate powder sintering and consolidation process. Increasing laser power resulted in an enhancement of specimen hardness due to improved particle coalescence. However, high laser powers (∼16.2–17.4 W) caused edge burning, thermal degradation, and curling. The issues emerged from excessive energy input into the layer and unbalanced surface tension forces, resulting from inadequate control over the mechanical strength of the layer's attachment to the build plate. Overall, they found that a laser power of 13 W with optimum values of ED (∼0.062–0.070 J/mm^2^) and scan speed (∼390 mm/s) maximized the mechanical properties of SLS‐printed PEI parts. Additionally, optimized powder flowability was achieved by precisely controlling the bed temperature throughout the process. However, for multilayer‐printed PEI samples, the optimal ED shifted slightly to 0.070 J/mm^2^, at a scan speed of 343.9 mm/s with the same laser power of 13 W [73]. The study revealed that the tensile strength of the SLS‐processed PEI samples increased gradually with ED, peaking at around 0.07 J/mm^2^ before significantly dropping. The highest tensile strength obtained was approximately 21 MPa, which is lower than that of FFF counterparts. However, the laser‐sintered samples were relatively free of surface irregularities, indicating a more homogeneous response to mechanical testing. Furthermore, porosity analysis showed that with increasing scan speed, the porosity level initially decreases but then increases, with the highest powder consolidation at 343.9 mm/s. Table 8 provides a summary of these ideal parameters and accompanying mechanical characteristics.

**TABLE 8:** Factors influencing selective laser sintering (SLS) of imide‐based (PEI, PI) polymers and corresponding optimal values.

Parameter	Optimal value(s)	Effects/Benefits	References
Energy density	0.062–0.070 J/mm^2^	Best laser sintering and mechanical properties	[73]
Laser power	13 W	Optimal sintering	[73]
Scan speed	343.9–390 mm/s	Optimal mechanical properties and consolidation	[73]
Bed temperature	Controlled to optimize powder flowability	Enhanced flowability and sintering quality	[73]
CO_2_ laser	60 W	Suitable for sintering PEI	[73]
Annealing temperature	Over 280°C for more than an hour	Necessary for crystallization and reduced warping	[50]
Crystallization kinetics	Slow crystallization kinetics	Reduced residual stress and warping	[50]
Tensile strength	21.9 MPa	Achieved with ED of 0.07587 J/mm^2^	[73]
Optimum single‐layer sintering ED	0.062 J/mm^2^ (13 W, 386.9 mm/s)	Best conditions for single‐layer sintering	[73]
Optimum multilayer printing ED	0.070 J/mm^2^ (13 W, 343.9 mm/s)	Best conditions for multilayer printing	[73]

#### 3.2.4 Critical Synthesis and Research Priorities for SLS of HPPs

Numerous studies have demonstrated the considerable potential of the SLS technique for 3D printing HPPs with relatively reduced material anisotropy and improved structural integrity compared to FFF. However, the existing conclusions raise apparently conflicting findings regarding process optimization, mechanical performance, density, powder morphology, crystallinity, bed temperature, and laser energy density (ED). For instance, several works demonstrated that increasing ED improved interlayer and interparticle coalescence through neck formation, thereby enhancing part density and mechanical performance. However, other studies reported that excessive ED facilitates polymer oxidation and thermal degradation, thereby affecting powder morphology, flowability, surface smoothness, and dimensional accuracy. Collectively, these results suggest that laser ED alone does not guarantee part integrity and mechanical performance. Therefore, it is crucial to understand the synergistic interactions among ED, powder morphology, powder flowability, scanning speed, and powder bed temperature, governing the balance between thermal degradation and appropriate particle diffusion.

A similar inconsistency appears in reported crystallization kinetics of sintered particles of HPPs. While the studies demonstrate that increasing the print bed temperature reduces thermal gradients and localized residual stress accumulation, excessive bed temperature and prolonged duration of particles may promote thermal degradation, powder aging, undesirable crystal growth, and reduce the reusability of unused powders. Therefore, an optimized processing window is essential for balancing crystallization behavior and powder‐bed temperature, which was often ignored in the reported literature.

Another critical challenge for SLS‐printed HPPs is to minimize the porosity content, which is not rigorously discussed in the existing literature. Unlike FFF, these microstructural defects in SLS are primarily governed by powder morphology, laser ED, incomplete particle diffusion, and particle size distribution (PSD), rather than inappropriate interlayer adhesion. Therefore, an optimization strategy between these factors is essential to mitigate these microstructural defects and improve print quality, part density, and mechanical performance.

Table 9 presents a cross‐study synthesis of the SLS technique for AM of HPPs with high resolution and reliability. Overall, the cross‐study synthesis suggests that the effectiveness of the SLS technique for HPPs is primarily governed by synergistic interactions between various print parameters and powder characteristics. Therefore, a comprehensive study is essential to developing optimum process‐structure‐property relationships for successful AM of HPPs using the SLS technique.

**TABLE 9:** Cross‐study synthesis of selective laser sintering (SLS) of high‐performance polymers.

Parameter	Key conclusion	Context–dependent observation	Unresolved challenge
Energy Density (ED)	Essential for complete powder fusion	Excess ED causes thermal degradation and structural distortion	Adaptive and ramp energy control
Bed temperature	Reduces internal residual stress and warpage	Excess temperature facilitates powder aging	Uniform crystallinity around the structure
Powder morphology	Spherical powder particles enhance density and flowability	Depends on particle size distribution (PSD)	Qualification standards for powder particles
Crystallinity	Improves structural integrity, stiffness, and heat resistance	Excess crystallinity reduces toughness	Controlling crystallization kinetics
Mechanical anisotropy	Relatively lower than FFF	Localized heat concentration may still create variability	Controlling a uniform thermal field and heat management system

### 3.3 Direct Ink Writing (DIW) Process

Direct Ink Writing (DIW) is a type of extrusion‐based AM technique, which facilitates room‐temperature 3D printing of various viscoelastic materials, including hydrogels, liquid crystal elastomers (LCEs), colloidal suspensions, and polymeric inks [110, 111, 112, 113]. A typical DIW process consists of two major components: a pressure‐driven extrusion head and a translational build platform (Figure 5a). This process is primarily recognized for its adaptability with versatile materials, as long as the precursor inks exhibit appropriate rheological properties and shear‐thinning behavior. Therefore, to make the inks printable with high shape retention capability after extrusion, rheological modification is a key prerequisite for successful DIW printing. By integrating various functional additives or fillers, the DIW process offers the freedom to tailor the ink rheology. For example, carboxylated cellulose nanocrystals (cCNCs), methyl cellulose (m‐cellulose), xanthan gum, or nanoclay are the most commonly used rheology modifiers [114, 115]. The precursor inks are usually made with viscosities between 10^2^ and 10^6^ mPa·s at an average shear rate of around 0.1 s^−^^1^ to provide reliable printability [116, 117, 118, 119], as illustrated by the ideal rheological behavior of a DIW ink (Figure 5a). Although the DIW process demonstrates its suitability for various biopolymers and soft materials, incorporating this technique to print HPPs is challenging due to their intrinsic material properties, such as poor solubility, poor flow rheology, and poor surface adhesiveness. Thus, a thorough study of ink rheology and optimization of printing conditions is required to facilitate the 3D printing of HPPs via DIW.

**FIGURE 5:**
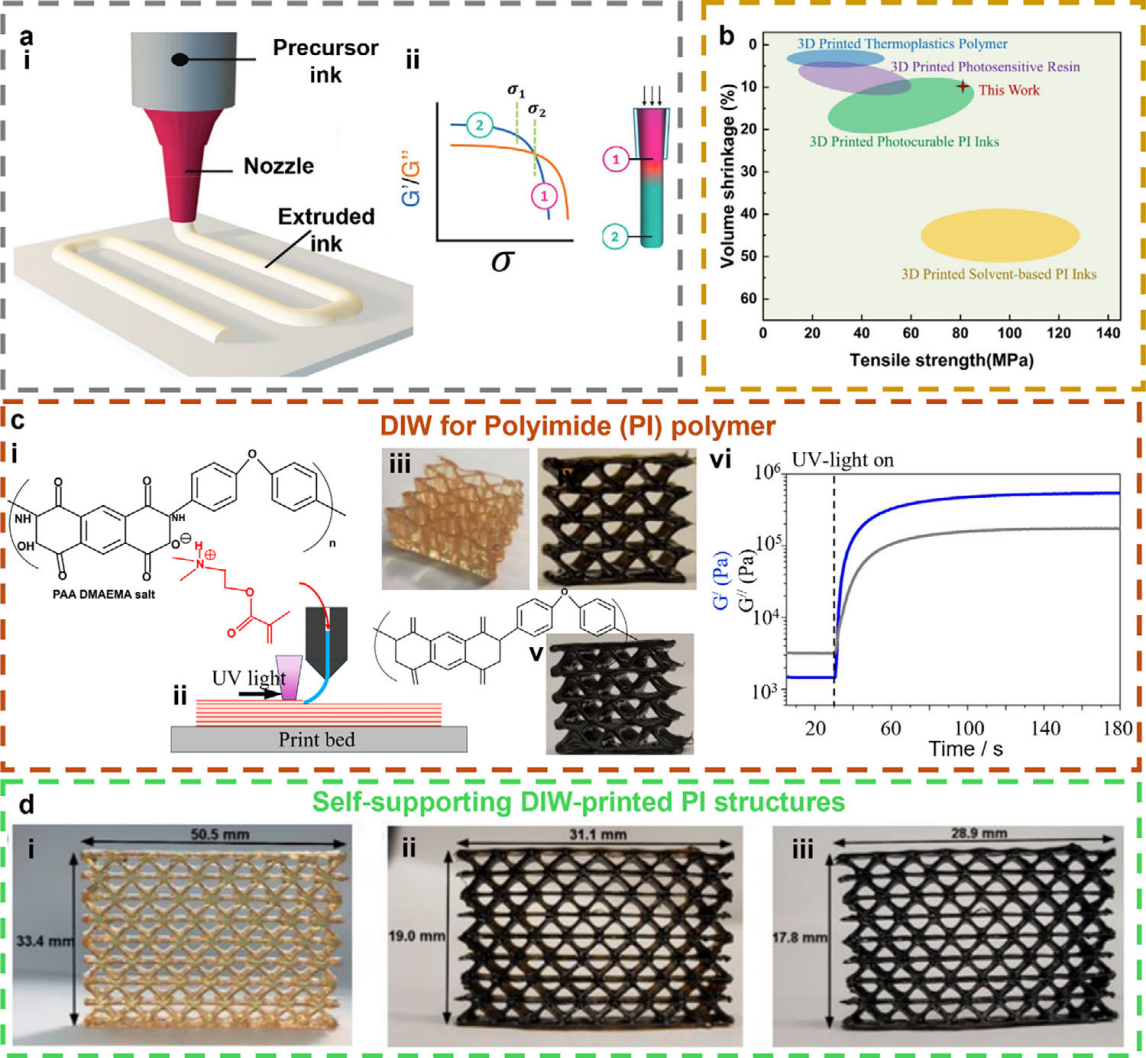
(a) Direct ink writing (DIW) of polyimide precursors and associated ink rheology and post‐processing. (i) Schematic representation of the DIW process, and (ii) Schematic color map of pressure‐driven flow of precursor ink and its rheology: changes in storage modulus (G′) and loss modulus (G″) with shear stress (σ). Reproduced (Adapted) with permission [116]. Copyright 2022, John Wiley and Sons. (b) Ashby plot showing the relationship between volume shrinkage (%) and tensile strength of UV‐DIW‐printed polyimide. Reproduced (Adapted) with permission [120]. Copyright 2024, Elsevier. (c) DIW printing scheme for polyimide (PI) polymer: (i) Appropriate ink formulations, (ii) UV‐assisted DIW AM technology, (iii) Printed self‐supporting 3D structure, (iv) Post‐processing of the printed parts for imidization with heat treatment for solvent removal, (v) Thermal treatment promotes conversion and imidization of the precursor system, yielding the final PI parts. Reproduced (Adapted) with permission [75]. Copyright 2018, American Chemical Society, and (vi) Rheology of the PI inks: moduli vs. time from the rheological measurements of the PI inks with a starting time of UV irradiation at 30 s. Reproduced (Adapted) with permission [75]. Copyright 2018, American Chemical Society. (d) Images of (i) organogel self‐supporting structure (as‐printed), (ii) Thermal curing in vacuum at 200°C, and (iii) Thermal imidization under a nitrogen environment at 400°C. Reproduced (Adapted) with permission [75]. Copyright 2018, American Chemical Society.

#### 3.3.1 DIW of Polyimide (PI)

The fabrication of complex PI structures through the DIW technique presents significant challenges, primarily due to the high viscosity and limited solubility characteristics of PI. Therefore, to create complex PI structures using the DIW technique, it is essential to develop PI ink with suitable rheology modifiers. Early attempts to print PI structures using the DIW technique encountered challenges, including poor ink rheology, layer separation, filament sagging, and poor shape fidelity. These challenges were primarily due to its high melt viscosity and insolubility in various solvents, making it difficult to formulate a suitable ink for DIW. To overcome these challenges, researchers employed various precursors to enhance solubility and utilized rheology modifiers to improve printability. To stabilize the deposited inks, researchers introduced in situ curing mechanisms before the imidization of the precursor ink. Despite these attempts, many challenges remain in the DIW of PI. These include dimensional instability for large green structures, precursor ink requiring post‐curing at high temperature (>300°C), printed structures being prone to cracking, and volume shrinkage during solvent removal. Figure 5b compares the volume shrinkage with tensile strength reported for DIW‐printed PI systems in the literature. In general, solvent‐based PI inks tend to undergo greater dimensional shrinkage due to solvent evaporation and the volume contraction associated with thermal imidization, whereas solvent‐free formulations can significantly mitigate these effects. For instance, Wei et al. reported a UV‐curable, solvent‐free PI ink that achieved less than 6% volume shrinkage during UV‐assisted DIW while maintaining a tensile strength of ∼75 MPa [120]. Accordingly, the Ashby plot highlights the ongoing challenge of balancing dimensional stability and mechanical performance, while also demonstrating that both objectives can be achieved simultaneously through appropriate ink design and processing strategies.

Several studies have demonstrated the processability and solubility of PIs with organic solvents to develop architected PI structures [75, 120, 121]. Rau et al. demonstrated the successful printing of a PI structure utilizing a custom‐built UV‐assisted DIW technique [75]. In this study, they used pyromellitic dianhydride (PMDA) and 4,4′‐diaminodiphenyl ether (ODA)‐based poly(amic acid) (PAA) as the precursor of PI to develop a DIW‐suited PMDA‐ODA PAA PI ink (Figure 5c). In situ curing of the subsequently deposited layers is essential to obtain self‐supporting PI structures. For DIW printing of photopolymer resins, the UV irradiation polymerizes the deposited layers. This in situ effective UV polymerization depends on several key factors. Some of these are the gel time and gel state modulus of the precursor ink, UV light intensity, printing speed, and UV light permittivity. Out of all these factors, the shorter gel time plays a vital role in ensuring shape fidelity and improved interlayer bonding. The gel state modulus plays an essential role in the DIW process, influencing shear‐thinning behavior and ensuring shape retention during material extrusion. Therefore, it is crucial to strike a precise balance between the gel time and gel state modulus for printing PI structures using a UV‐assisted DIW process. They synthesized a customized PI precursor solution to obtain a balance between the modulus at the gel state and the gel time under UV exposure. In this synthesis process, they modified the PAA precursor ink by adding an amino‐functional methacrylate photoresin, 2‐(dimethylamino)ethyl methacrylate (DMAEMA), to formulate a PAA DMAEMA salt solution (Figure 5c). The synthesized PAA DMAEMA resin shows a high viscosity (zero‐shear rate viscosity of 770 Pa·s), high gel state modulus or storage modulus (G′ = 5 × 10^5^ Pa), shear‐thinning behavior, and shorter gel time (∼1.7 s) (Figure 5c(vi)), making it suitable for DIW printing with effective in situ photopolymerization. Finally, the printed and subsequently UV‐cured PAA DMAEMA structure exhibits self‐supporting deposition and good shape retention potential. The printed structure undergoes solvent removal and drying at 400°C to induce imidization, leading to the formation of aliphatic crosslinks between PAA and DMAEMA. The imidized structure exhibits an isotropic shrinkage of ∼45% and a weight loss of ∼83%. In this work, they obtained an imidized PI structure with good shape fidelity and higher PI solid loading of ∼17% (Figure 5d). These results highlight how the UV‐assisted DIW process, photocurable resin synthesis, and adjustments to other parameters can improve print quality and yield self‐supporting PI structures.

In another study, Xue et al. utilized a freeze‐assisted DIW (FADIW) technique to 3D print PI with controlled aerogel structures for applications in thermal management systems. They also used PAA (8 wt% solid content) as the precursor ink (Figure 6a(i)) [42]. They utilized a Joule‐heated syringe at a temperature of 40°C to maintain ink rheology, reduce extrusion pressure, enhance print resolution, and improve solvent management. Additionally, partial gelation on a temperature‐controlled substrate (−5°C) helps to maintain the structural integrity and print large PI structures. By controlling the substrate temperature, the microstructure of printed PI aerogels can also be controlled to optimize the thermal insulation property. The final imidized structures were obtained through the subsequent lyophilization and post‐thermal treatment processes (300°C), which exhibited excellent printability, reduced weight, and significant load‐bearing capacity (Figure 6a(ii‐v)). Qin et al. also conducted a similar study utilizing the DIW technique to print PAA ink [122]. They formulated the desired PAA salt hydrogels by mixing 6.99 g of 2‐dimethylaminoethanol (DMEA) and 20.00 g of PAA with 73.01 g of water to get a PAA salt solution with 20 wt% solid content. The printed PI structures, after being imidized at 250°C, exhibit improved thermal stability, dimensional stability, and mechanical properties. These results further highlight the processability and printability of PAA precursor inks for DIW. However, the significant dimensional shrinkage (>45%) after imidization of the printed PI structures impedes the potential of the DIW technique. To enhance the dimensional accuracy and mechanical performance of printed polyimide (PI) structures, Guo et al. employed a UV‐assisted DIW approach that incorporated a two‐step curing process consisting of UV curing followed by thermal imidization [123]. This strategy effectively reduced shrinkage during imidization while improving the mechanical properties of the final printed parts. Like other studies, they used PAA as the PI precursor but modified it using the UV‐curable functional group hydroxyethyl methacrylate (HEMA). They synthesized the HEMA‐grafted PAA ink (PAA‐*x*HEMA) at various mol% of HEMA (20, 30, 40, and 50). They introduced a reinforcing multifunctional crosslinker, trimethylolpropane triacrylate (TMPTA), to the precursor PAA‐xHEMA ink to minimize the dimensional shrinkage of the printed PAA‐xHEMA PI structures. An aprotic polar solvent N, N‐dimethylformamide (DMF), was mixed into the precursor ink as a rheology modifier. The slow evaporation of DMF solvent enhances printability by enabling crack‐free solidification of the printed structures. Furthermore, post‐thermal imidization with TMPTA facilitates rapid polymerization and increases crosslinking density, resulting in enhanced dimensional stability. Together, this distinctive synthesis of the precursor PAA‐xHEMA ink optimizes the printability, fast curing, and shape fidelity with negligible dimensional shrinkage (∼6%) after post‐thermal imidization process at 300°C (Figure 6b). The high‐precision printed PAA‐xHEMA PI structures (spring and enameled wire) further highlight the processability of PI precursors utilizing the DIW technique with negligible dimensional shrinkage (∼6%), filament sagging, improved structural integrity, and improved mechanical properties (Figure 6c).

**FIGURE 6:**
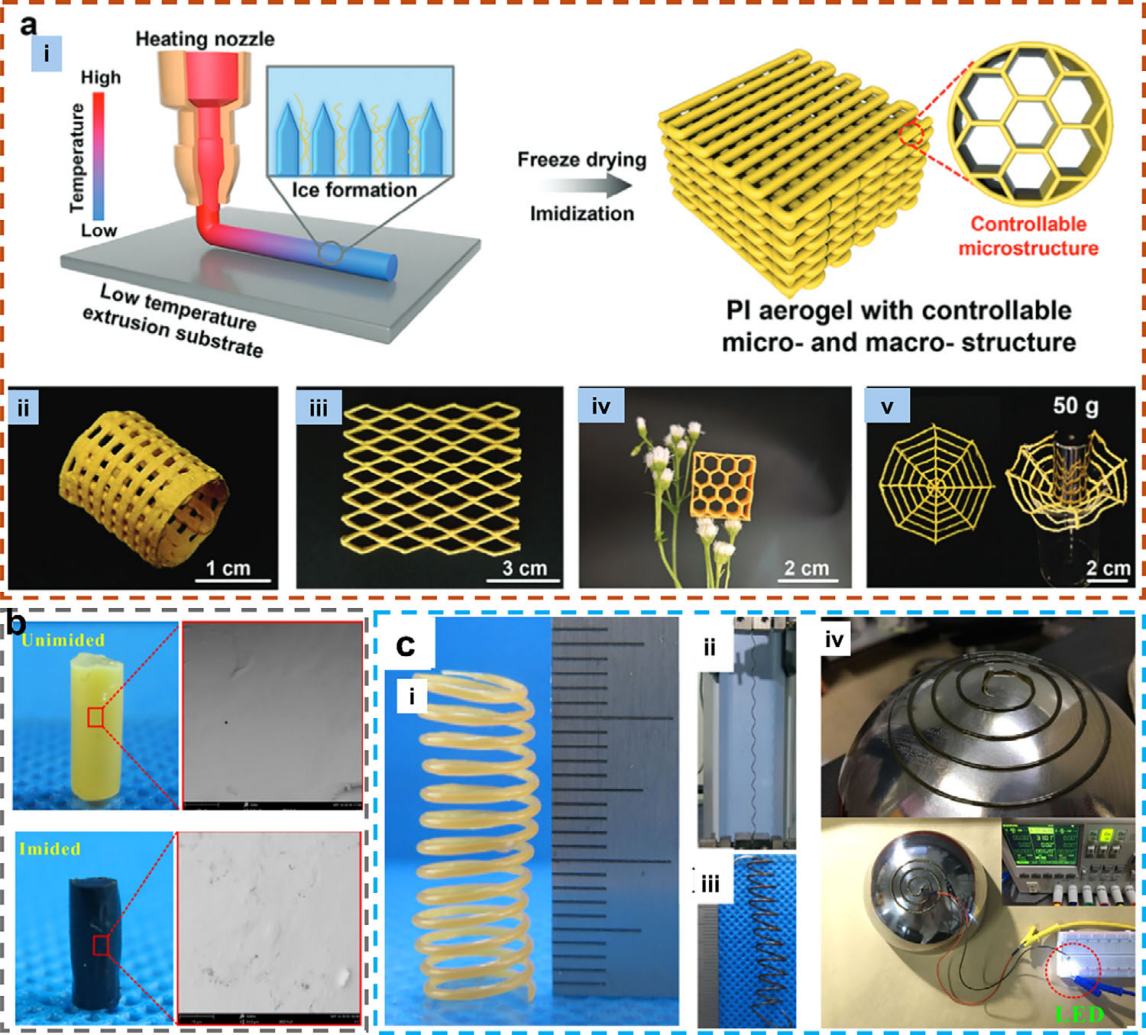
DIW printing of polyimide (PI) precursor inks. (a) Freeze‐assisted DIW (FADIW) printing process of PI aerogels, where pyromellitic dianhydride (PMDA) and 4,4′‐diaminodiphenyl ether (ODA)‐based poly(amic acid) (PAA) aqueous solution (8 wt% solid content) acts as the PI precursor: (i) DIW scheme inducing in situ freezing on a temperature‐controlled substrate to control the morphology of the printed structure. By controlling the temperature of this substrate, the growth and size of ice crytals are maintained to obtain the desired pore size for optimal thermal insulation properties; (ii, iii) FADIW printed PI structures; (iv) FADIW printed lightweight (0.04 g/cm^3^) PI honeycomb structure that a flower can withstand the weight; and (v) Printed spider web PI structure and its capacity to withstand weights of up to 50 g, indicating the load‐bearing capacity of the PI structures. Reproduced (Adapted) with permission [42]. Copyright 2024, John Wiley and Sons. (b,c) DIW‐printed PI structures based on the PAA aqueous solution as the PI precursor, which is modified with UV‐curable functional group hydroxyethyl methacrylate (HEMA) to provide rapid polymerization under UV irradiation: (b) The printed PI structure shows excellent dimensional stability during the imidization process at high temperatures (negligible volume shrinkage ∼6%), and (c) DIW‐printed mechanical spring, indicating excellent self‐supporting structure: (i) before tensile stretching, (ii, iii) after 20 cycles tensile stretching at 200% strain, and (iv) Multi‐extrusion nozzle DIW printed core‐shell structure enameled wire on a curved surface with high dielectric property, where carbon paste in the core acts as the conductive wire and PI in the shell acts as the insulation layer. Reproduced (Adapted) with permission [123]. Copyright 2019, John Wiley and Sons.

#### 3.3.2 DIW of Polytetrafluoroethylene (PTFE)

3D printing of PTFE using the DIW technique poses significant challenges, owing to the high melt viscosity, adverse ink rheology, and the need for appropriate formulations of PTFE ink, as well as poor shape retention after extrusion. Additionally, another significant challenge is printed PTFE structures typically require post‐thermal processing at high temperatures to ensure complete sintering and coalescence across adjacent deposited regions, thereby enhancing interlayer adhesion. Therefore, optimizing the curing temperature is crucial for preventing thermal degradation of the printed PTFE structures [124]. Despite these challenges, multiple studies have demonstrated the processability and printability of PTFE ink using DIW by adjusting the PTFE formulations, ink rheology, printing parameters, and post‐processing conditions.

PTFE ink can be formulated using concentrated PTFE paste or colloidal PTFE nanoparticle dispersions, with rheology modifiers like gellan gum (GG) or binders added to ensure the shape fidelity of the deposited ink. Utilizing surfactant‐stabilized PTFE nanoparticle (200 nm diameter) dispersions (60 wt%) and GG, Jiang et al. obtained DIW‐printed PTFE structures [76]. They also demonstrated that the mechanical properties of the printed PTFE structures are tailorable by tuning the ink formulations and other processing parameters. As PTFE is highly hydrophobic, they used ionic surfactants poly(oxy‐1,2‐ethanediyl) and α[3,5‐dimethyl‐1‐(2‐methylpropyl) hexyl]‐ω‐hydroxy in the PTFE dispersion to stabilize the nanoparticles in water (Figure 7a(i)). To achieve suitable shear‐thinning behavior of the aqueous PTFE nanoparticle dispersion, they prepared different ink formulations by adding various rheology modifiers and binders, such as xanthan gum, gellan gum (GG), and agar (Figure 7a(ii)). Among these modifiers, they found that the ink with GG content (1.5 wt%) provides the best shear‐thinning behavior to the PTFE dispersion. Before adding GG, the surfactant‐stabilized PTFE dispersion was heated at 50°C to control the ink rheology through polymer‐colloidal interactions. This preheating also helps the GG to be hydrated and dissolved uniformly into the PTFE dispersion. Therefore, the GG dissolved in the PTFE dispersion shows sol‐gel‐like and shear‐thinning behavior, making it suitable for DIW printing with a 0.8 mm extrusion nozzle (Figure 7a(iii)). Additionally, GG forms physical crosslinking through hydrogen bonding within the printed PTFE structures, providing robust structural integrity and enhancing mechanical properties. They used multistage heating cycles to remove the organic solvents from the printed green structures at ∼260°C and impart structural stability by a sintering cycle at ∼380°C (Figure 7a(iii)). The sintered PTFE honeycomb structures (Figure 7a(iv)) demonstrate smooth surface morphology, super‐hydrophobicity (contact angle beyond 120°), and uninterrupted printability of the GG/PTFE ink. Figure 7a(v) indicates the uniform distribution of fibrillar microstructure within the printed structures after the sintering cycle. Therefore, the sintering of the printed structures is crucial for fusing and coalescing the PTFE nanoparticles throughout the structures, which minimizes porosity, increases hydrophobicity, and improves mechanical properties. The printed PTFE structures also exhibit strong chemical inertness under various temperature conditions in extreme acidic (1 M hydrochloric acid, HCl) and basic (3 M sodium hydroxide, NaOH) environments (<3.0% mass loss after 7 days) (Figure 7a(vi)). Together, these results demonstrate that PTFE nanoparticles can be effectively processed using the DIW technique, enabling controlled microstructure, adjustable mechanical properties, hydrophobicity, and chemical inertness.

**FIGURE 7:**
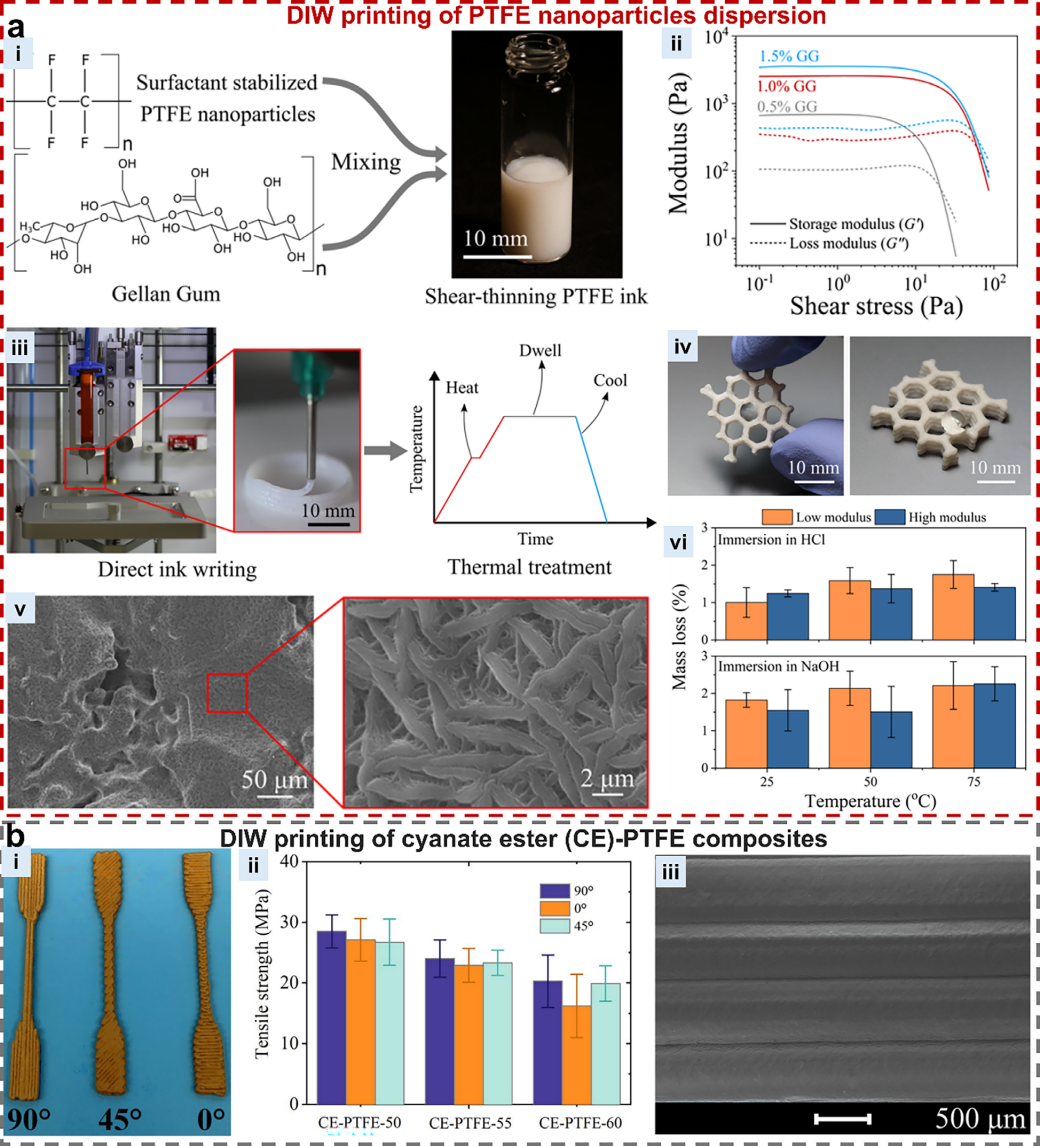
DIW printing of PTFE‐based inks using different rheology modifiers. (a) PTFE nanoparticle dispersion‐based scheme for DIW: (i) Ink formulation and molecular structures of surfactant‐stabilized PTFE nanoparticles (diameter: ∼200 nm) dispersion with gellan gum (GG) as a rheology modifier; (ii) Storage modulus (G′) and loss modulus (G″) of the PTFE/GG inks for different weight contents of GG under shear stress. The ink with 1.5 wt% GG shows excellent rheological properties for the DIW process; (iii) Two‐step fabrication scheme of PTFE/GG ink: DIW printing with a 0.8 mm extrusion nozzle and multistage post‐thermal treatments to remove solvents at ∼260°C and induce sintering for thermal fusion and coalescence of the PTFE nanoparticles at ∼380°C; (iv) Printed honeycomb structures, demonstrating smooth surface morphology, strong hydrophobicity (contact angle exceeding 120°), and uninterrupted printability of the GG/PTFE ink; (v) An SEM image of a high modulus tensile sample indicates the uniform distribution of fibrillar microstructure within the printed structures after the sintering cycle at ∼380°C; and (vi) Characterization of the printed structures for chemical inertness under strong acidic (1 M hydrochloric acid, HCl) and basic (3 M sodium hydroxide, NaOH) environments at varying temperatures for 7 days. Reproduced (Adapted) with permission [76]. Copyright 2019, American Chemical Society. (b) DIW printing of bisphenol‐E‐type cyanate ester (CE) with PTFE micropowder (powder size 5 µm), where PTFE micropowder acts as a thixotropic agent to modify the ink rheology: (i) DIW‐printed tensile samples using the PTFE/CE ink at different print orientations (90°, 45°, and 0°); (ii) Tensile strengths of the printed PTFE/CE composite inks at various PTFE loadings (50%, 55%, and 60 wt%) and different print orientations (90°, 45°, and 0°); and (iii) Scanning electron microscopic image of the deposited filaments for the PTFE/CE composite ink with 50 wt% PTFE content (CE‐PTFE‐50), indicating uniform and continuous deposition of the composite ink with excellent interlayer adhesion and negligible void content. Reproduced (Adapted) with permission [125]. Copyright 2024, Springer Nature.

In another work, Zhao et al. utilized PTFE micropowder (particle size ∼ 5 µm) as a rheology modifier within bisphenol‐E cyanate ester (CE) thermoset resin [125]. The incorporation of PTFE micropowder into the CE monomer imparts gel‐like behavior with good shape retention capability, making it suitable for DIW. In addition, the printed structures exhibit excellent print quality and mechanical properties (Figure 7b(i‐ii)). The composite ink also shows continuous deposition through the extrusion nozzle with excellent interlayer adhesion and minimal void content (Figure 7b(iii)). Compared to the mechanical properties of PTFE nanoparticles/GG structures printed by Jiang et al., the CE/PTFE specimens exhibit higher mechanical properties along three different print orientations [76]. The CE monomer establishes chemically robust crosslink networks with PTFE micropowder during the post‐thermal treatment. In contrast, the GG functions as a temporary binding agent, providing limited physical strength through hydrogen bonding. Also, the CE network enhances the dimensional (volumetric shrinkage ∼2.8%) and thermal stability of the printed structures during post‐thermal processing. However, at higher curing temperatures, the printed CE/PTFE structures may exhibit reduced mechanical toughness due to rigid chemical bonding and limited chain mobility, restricting their usage for extreme environmental conditions. Therefore, further studies are required to optimize PTFE ink formulations, DIW process parameters, and post‐thermal curing conditions to maximize the material properties of DIW‐printed PTFE structures.

#### 3.3.3 Critical Synthesis and Research Priorities for DIW of HPPs

In comparison with FFF and SLS, the development of the DIW technique for HPPs is still in its early stages. As a result, the existing studies are relatively fragmented, most of which are based on different rheological modifiers, tailored precursor formulations, and different post‐processing approaches. A careful review of these studies suggests that differences in printing hardware do not primarily cause contradictory findings. Instead, they largely stem from variations in rheological properties, precursor chemistry, post‐print solvent evaporation behavior, and curing kinetics or imidization procedures. Table 10 presents a cross‐study synthesis framework of the DIW technique for 3D printing of HPPs.

**TABLE 10:** Cross‐study synthesis of the direct ink writing (DIW) technique of high‐performance polymers.

Parameter	Key conclusions	Context–dependent observation	Unresolved challenge
Ink rheology	Shear‐thinning and shape retention are crucial for printability	Optimum rheology depends on nozzle size and shape, and solid loading	Standardized rheological design criteria
Shape fidelity	High yield stress ensures dimensional stability	Excess yield stress restricts printability and interlayer coalescence	Balancing interlayer bonding, printability, and shape fidelity
Solvent removal	Drying and imidization govern volumetric shrinkage, residual stress, and crack formation.	Highly dependent on solvent system, part geometry, heating rate, and heating environments	Crack‐free curing kinetics and part densification
Post‐processing	Thermal treatment promotes polymer diffusion and improves structural integrity.	Excessive heating causes oxidation, microcracking, and structural distortion	Optimized curing kinetic protocols

Several studies reported that the optimization between nozzle diameter, extrusion pressure, and print speed is essential to improving print fidelity. Conversely, others claimed that appropriate ink rheology with suitable precursors is much more important than the print parameters. Another concern is regarding the modifiers, sacrificial binders, and the type of solvents. Although these are inevitably important for DIW, these additives eventually need to be removed from the 3D‐printed structures through various drying and solvent removal approaches, which often introduce microcrack formation, residual stress accumulation, and substantial volumetric shrinkage. The variations in the ink formulations, print parameters, and post‐processing may reflect conflicting conclusions within the reported studies.

Similarly, the conclusions regarding shape fidelity and yield stress still have some conflicts. While the yield stress ensures appropriate viscoelastic behavior and immediate shape retention after extrusion, which is an ink characteristic, the printability depends on other factors, like nozzle geometry, nozzle diameter, nozzle length, print speed, and print geometry. Also, excess yield stress can result in discontinuous ink flow, insufficient volumetric flow rate, poor interlayer material fusion, and weaker interlayer coalescence. Conversely, lower yield stress promotes flowability, but the printed parts may suffer from structural collapse unless in situ polymerization or curing is performed carefully. These all suggest that the yield stress alone cannot ensure the shape fidelity and smooth printability of the ink; instead, the other geometric and printing factors should be considered for developing optimal process‐structure‐property relationships. Overall, a synergistic optimal balance between the ink characteristics, precursor chemistry, print parameters, and post‐processing is essential to ensure successful AM of HPPs using the DIW technique.

## 4 Process‐Structure‐Property Relationship and Extreme‐Service Qualification

The successful deployment of additively manufactured HPPs in extreme environments is governed by a sequence of interconnected factors that begins with polymer chemistry and AM processing, continues through defect formation and microstructural development, and ultimately determines property retention under service conditions. This section first maps representative extreme‐service conditions to the material properties and AM‐related defects that govern component performance. It then outlines the qualification requirements needed for safety‐critical deployment, identifies transferable governing principles across FFF, SLS, and DIW, and integrates these relationships into a unified process‐structure‐property framework. Finally, the long‐term retention of properties under combined thermal, mechanical, chemical, and environmental exposure is considered.

### 4.1 Environmental Mapping, Failure Mechanisms, and Qualification Requirements

HPP components used in aerospace, automotive, marine, defense, energy, and nuclear applications may be exposed to elevated temperatures, cyclic loading, creep, impact, radiation, moisture, pressure, and chemically aggressive environments. The required material attributes therefore depend strongly on the intended service conditions. For example, high‐temperature applications require appropriate thermal transitions, thermo‐oxidative stability, and dimensional stability, whereas chemically aggressive environments require resistance to solvents, acids, bases, and salts. Structural applications additionally require adequate specific strength, toughness, fatigue resistance, and robust interlayer load transfer. Therefore, a systematic mapping of service stressors to material attributes is essential before AM‐route selection and qualification. Table 11 summarizes representative extreme‐service conditions, the material attributes they impose, and HPP systems identified within this review as candidate material platforms.

**TABLE 11:** Mapping of extreme‐service conditions to required material properties and candidate high‐performance polymers.

Extreme service conditions	Required material properties	Representative HPP candidates
High‐temperature exposure (>250°C)	High T_g_, T_m_, and thermo‐oxidative stability, and dimensional stability	PEEK, PEKK, PI
Aggressive chemicals/solvents	Low surface energy, chemical inertness, and resistance to solvents, acids, bases, and salts	PTFE
Mechanical/impact loading (aerospace)	High specific strength, toughness, fatigue resistance, and interlayer integrity	PEI, reinforced PAEKs
Radiation exposure (space/nuclear)	High shielding capability and retention of mechanical integrity	PEEK‐tungsten and PEEK‐boron composites

#### 4.1.1 Mapping Stressors to AM Route and Defect Formation

The performance and long‐term reliability of additively manufactured HPPs are intrinsically governed by the interplay between service‐induced stressors and process‐induced defects. Each AM route generates a distinct thermal history, interfacial architecture, and defect population; therefore, the selected manufacturing process strongly influences the subsequent degradation and failure mechanisms under extreme operating conditions. For instance, in FFF, semicrystalline HPPs typically require high melt‐extrusion temperatures (∼400°C), which create steep thermal gradients between the extrusion nozzle and the surrounding environment, facilitating rapid melt solidification and restricting polymer‐chain diffusion kinetics across adjacent fused filaments. Consequently, weak interlayer adhesion at the interface forms, leading to residual stress accumulation, interlayer delamination, and dimensional warpage [15, 51]. Under cyclic loading, thermal exposure, or impact, these interfaces can act as preferred sites for crack initiation and propagation. In SLS, incomplete powder fusion often causes entrapped air and voids and localized stress concentration sites in SLS‐printed parts. Consequently, the resulting structures, containing undesired porosity, become structurally challenging for marine, pressure‐bearing, or other load‐bearing applications requiring defect tolerance. Nevertheless, compared with FFF, SLS generally produces parts with more homogeneity and reduced anisotropy, making it an attractive AM route for many demanding applications where structural reliability is critical [73, 93, 102]. DIW technique, particularly for polyimide (PI) precursors, presents a different set of process‐induced limitations. For instance, large dimensional shrinkage (∼45%) during imidization and solvent removal can introduce micro‐cracks and residual stresses, reducing structural integrity and mechanical performance unless post‐processing is systematically optimized [123]. Thus, although DIW avoids high‐temperature melt extrusion during deposition, the final structure remains strongly dependent on the subsequent conversion and densification processes. Overall, these examples demonstrate that the selection of an appropriate AM route can not only influence the printability but also the intensity of processing‐induced microstructural defects, durability, and mechanical performance of the 3D‐printed HPPs for the target‐specific high‐temperature applications.

#### 4.1.2 Qualification Requirements and Certification Pathways

To facilitate the practical deployment of 3D‐printed HPPs in safety‐critical and extreme‐service applications, a structured qualification framework is indispensable beyond process optimization and material selection. Figure 8 represents a conceptual service‐driven framework for AM of HPPs for extreme environment applications, highlighting a representative qualification roadmap that encompasses material qualification, process qualification, structural qualification, and service or digital qualification. Material qualification should first establish whether the selected HPP possesses the intrinsic thermal, mechanical, chemical, flammability, and environmental characteristics required for the target application. For example, compliance with flame, smoke, and toxicity (FST) standards (e.g., FAR 25.853) needs to be maintained for aerospace applications [126]. Process qualification should then be introduced to verify reproducibility with minimized porosity, print quality, and mechanical performance of the 3D‐printed parts using the selected AM route. Statistical significance and in situ monitoring can also be incorporated into the system for identifying interlayer defects, process improvement, dimensional viability, and reliability. Structural qualification needs to be developed to assess the quality and reliability of the 3D‐printed HPP structures with appropriate mechanical and functional characterizations, including tensile, flexural, fatigue, fracture, creep, and impact testing, along with the material anisotropy and interlayer strength. Finally, service qualification should establish performance retention under representative environmental conditions. Accelerated aging, thermal cycling, chemical exposure, radiation, pressure, humidity, and combined mechanical–environmental testing may be required. Emerging digital tools, including thermal imaging, embedded sensors, structural health monitoring, digital twins, and machine‐learning‐assisted damage detection, can supplement conventional qualification by enabling real‐time condition assessment and predictive maintenance of components operating in inaccessible or safety‐critical locations [127, 128, 129, 130, 131]. Collectively, this staged qualification framework demonstrates an effective and strategic roadmap for suitable material selection, AM route selection, defect mitigation, process optimization, and environmental durability, supporting the reliable deployment of 3D‐printed HPP structures in target‐specific high‐temperature applications.

**FIGURE 8:**
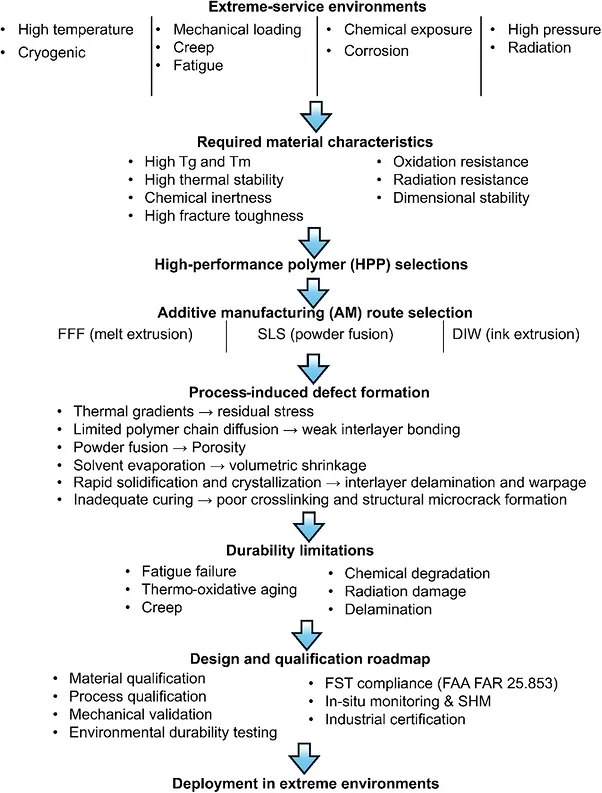
Service‐driven conceptual framework for 3D printing of high‐performance polymers (HPPs) for extreme environments.

### 4.2 Governing Principles for Additive Manufacturing of HPPs

Although a considerable body of literature has emerged on the AM of HPPs, many reported observations remain fragmented, process‐specific, and occasionally contradictory. Differences in polymer grade, equipment configuration, thermal boundary conditions, specimen geometry, and testing procedures make it difficult to transfer an optimum parameter set directly from one system to another. Therefore, broader governing principles are needed to interpret the recurring relationships among thermal history, rheology, crystallization, interlayer diffusion, porosity, dimensional stability, and mechanical anisotropy. Establishing these principles separates general mechanisms from process‐specific observations and provides the basis for predictive design under extreme‐service constraints. Table 12 condenses these recurring relationships into four governing principles, showing how each mechanism creates a corresponding processing‐performance trade‐off. Together, these principles provide the mechanistic basis for the unified selection framework developed in the following section.

**TABLE 12:** Governing principles, mechanistic insights, and associated processing‐performance trade‐offs in additive manufacturing of high‐performance polymers.

Governing principle	Mechanistic basis	Key trade‐off
Thermal balance	Interlayer adhesion requires a balance between rapid solidification for shape fidelity and slow cooling for chain diffusion [51, 59, 132]	Higher temperatures improve bonding but may cause oxidation, degradation, charring, residual‐stress accumulation, and dimensional distortion.
Reinforcement–porosity relationship	Fibers and particulate fillers can improve stiffness, thermal stability, dimensional control, and functional performance [133]	Reinforcements may restrict material flow and interlayer diffusion, increase interfacial porosity, and reduce impact, fatigue, and fracture resistance.
Rheological control	Melt‐based AM requires adequate flow for extrusion or particle coalescence together with sufficient melt strength for shape retention. DIW additionally requires shear‐thinning behavior, appropriate yield stress, and rapid recovery after deposition [123]	Improved flow enhances printability and bonding, whereas excessively low viscosity reduces shape stability; chemical modification may improve extrusion but complicate curing and imidization.
Crystallization Control	Thermal history and cooling rate determine crystallization kinetics and therefore stiffness, thermal resistance, shrinkage, and dimensional retention in semicrystalline HPPs [54]	Increased crystallinity may improve thermal and mechanical performance; however, rapid or nonuniform crystallization can promote warpage, residual stress, and structural heterogeneity.

These trade‐offs are not independent; rather, thermal conditions, rheology, reinforcement, and crystallization interact throughout the printing sequence. This interdependence motivates a framework that links material selection and processing decisions directly to the final service requirements.

### 4.3 Unified Selection Framework for Additive Manufacturing of HPPs

The governing principles identified above describe recurring physical relationships; the present framework integrates them into a practical material‐to‐service selection sequence. It links polymer chemistry and thermal transitions to feedstock form and rheology, maps these characteristics to a compatible AM route and processing window, predicts the resulting morphology and defects, and finally evaluates whether the retained properties satisfy the target service environment. For example, the same rigid aromatic chemistry that supports thermal stability can increase melt viscosity, restricting nozzle flow and interlayer diffusion; increasing thermal input may improve consolidation but also raises the risk of degradation or charring [22, 27, 51, 85]. This sequence therefore provides a direct bridge from the governing principles in Table 12 to the process–structure–property framework summarized in Figure 9.

**FIGURE 9:**
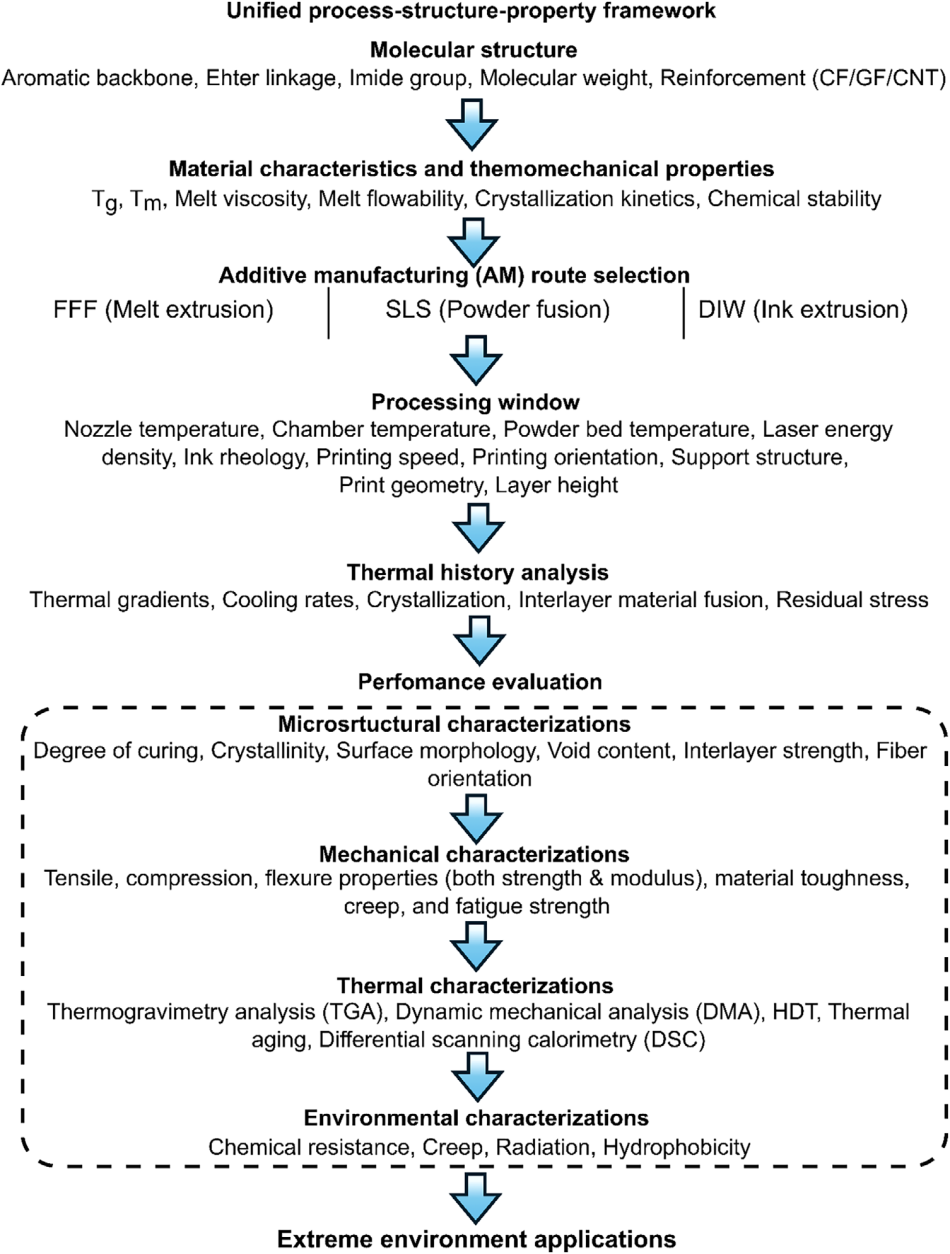
Unified process‐structure‐property framework linking HPP chemistry, thermomechanical properties, AM route and processing window, thermal history, microstructure, performance, and extreme‐environment applications.

Within this selection sequence, it is also essential to establish a unified thermal‐processing framework capable of correlating the dominant material characteristics and print parameters with final part performance. Given the strong interdependence between thermal history, crystallization kinetics, residual stress evolution, and interlayer diffusion in HPPs, a unified thermal‐processing framework helps select a strategic pathway for 3D printing of HPPs starting from suitable materials to the final targeted applications. In this context, Figure 9 provides the overall process‐structure‐property flow, while Table 13 translates that framework into material‐ and route‐specific processing windows, defect mechanisms, and service‐performance outcomes. Accordingly, the framework progresses from material and process selection to performance evaluation and, ultimately, target extreme‐environment applications.

**TABLE 13:** Material‐to‐service comparison of processing windows, defect mechanisms, anisotropy, dimensional stability, and extreme‐service performance across HPP additive manufacturing routes.

Material Class	Preferred AM route	Critical processing window	Dominant defect mechanism	Impact on porosity	Impact on anisotropy	Dimensional stability	Service performance
PEEK, PEKK [51, 97, 101]	FFF, SLS	Narrow: requires high chamber/nozzle temperature, controlled cooling	Incomplete interlayer fusion, thermal degradation, and residual stress	Moderate: highly dependent on bead overlaps and melt viscosity	High: Z‐axis strength is limited by polymer chain diffusion across the layer interface	Lower: High risk of warping due to differential shrinkage during crystallization	Excellent: High mechanical strength and fatigue resistance if processed correctly
PEI [134, 135]	FFF	Broad: no melting point; remains processable over a wider temperature range above T_g._	Layer‐induced anisotropy	Low: Smooth flow typically results in higher density (lower porosity)	Moderate: Lower than semicrystalline but still directional due to layer lines	Higher: Minimal shrinkage since there is no phase change (crystallization)	Balanced: Good strength and flame/smoke/toxicity (FST) ratings for aerospace
PTFE [46, 76]	DIW / Sintering	Ink rheology and post‐annealing	Volumetric shrinkage during drying and densification	High: Controlled porosity is often an inherent feature of the sintering process	Low: Post‐processing sintering helps promote more isotropic properties	Moderate: Subject to significant shrinkage during the sintering/densification phase	Specialized: Superior chemical inertness and low friction; lower mechanical load capacity
HPP composites (e.g., CF‐PEEK) [136]	FFF, SLS	Thermal management, fiber shearing, fiber dispersion, and fiber alignment	Fiber–matrix interfacial voids	Moderate: voids can form at the fiber‐matrix interface.	Variable: SLS is more isotropic than FFF, but fiber orientation can still cause bias	High: carbon/glass fibers act as anchors, significantly reducing thermal warping.	Excellent: high stiffness and thermal stability for structural replacements

Overall, the existing literature suggests that the performance of 3D‐printed HPP structures cannot be considered reliable and reproducible unless the synergistic contribution of material chemistry, material flow behavior, crystallization kinetics, thermal history, and manufacturing techniques is evaluated carefully. While the impact of each factor differs across the AM techniques, the underlying processing–structure–property relationship remains consistent. This integrated framework provides a reliable foundation for the selection of target‐specific materials, a suitable AM route, and optimized processing parameters, ensuring the performance and reliability of 3D‐printed HPP structures for desired service environments.

### 4.4 Property Retention Under Extreme‐Service Conditions

Beyond preliminary mechanical performance, property retention under prolonged exposure to extreme environments is one of the primary concerns for 3D‐printed HPPs. Existing literature has only demonstrated the 3D‐printability and short‐term use at elevated temperatures of a few representative HPPs, while research on their property retention under extreme service conditions for prolonged use remains scarce. This represents one of the primary research gaps limiting widespread qualification for industrial use of 3D‐printed HPPs. Hence, it is essential to understand their applicability, property retention, and structural integrity by considering the synergistic effects of creep, fatigue, thermo‐oxidative aging, and environmental effects.

## 5 Applications, Benchmarking, and Technology Readiness

Additively manufactured HPPs have gradually become popular in a myriad of demanding applications (Figure 10). This section evaluates demonstrated uses of additively manufactured HPPs in aerospace, automotive, defense, marine, energy, and radiation‐shielding systems and then distinguishes proof‐of‐concept demonstrations from qualified or deployment‐ready technologies. Particular attention is given to components produced from PEI, PAEK, PAI, PI, and PTFE, and the emphasis is placed on function, retained performance, benchmarking, and technology readiness.

**FIGURE 10:**
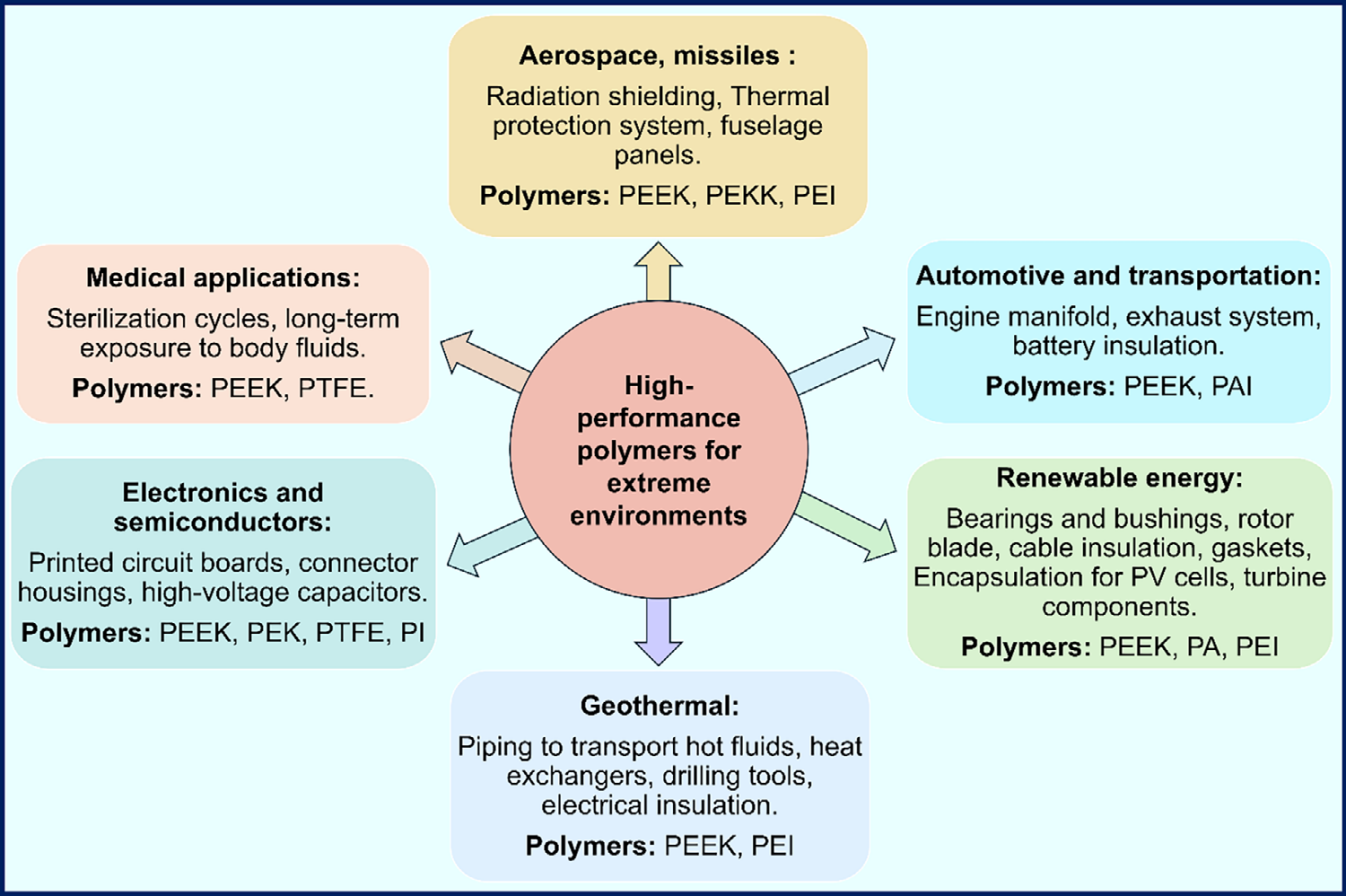
Representative high‐performance polymers and their applications in extreme‐service environments.

### 5.1 3D‐Printed HPPs in the Aerospace Industry

In aerospace applications, high strength‐to‐weight ratio, high thermal stability, and flame, smoke, and toxicity (FST) compliance are required, which makes HPPs such as PEI, particularly ULTEM 1010 and ULTEM 9085, a desirable material [135, 137]. The FFF technique remains the most prevalent fabrication process for PEI [15, 18]. Stratasys has led the aerospace industry by utilizing ULTEM 9085 to manufacture flight‐certified parts for the Airbus A350 XWB that minimize weight and lead time while meeting regulatory compliance (Figure 11a(i‐ii)) [65]. This achievement demonstrates the progress of the FFF process for functional and structural components under rigorous certification regulations. Similarly, the ULA achieved a 57% cost savings by substituting only 16 different components made with ULTEM 9085 for more than 140 heavy metallic components in its ducting system for the Environmental Control System (ECS) [12]. NASA has demonstrated how customized reinforcement enhances the performance of 3D‐printed parts by using chopped CF‐reinforced PEIs, both ULTEM 9085 and ULTEM 1010, for engine covers (Figure 11b(i)) and acoustic liners (Figure 11b(ii)). In comparison to injection‐molded components, the retention of tensile strength by 84% and stiffness by 64% in FFF‐printed 10% CF‐reinforced ULTEM 1010 indicates a level of structural equivalence (Figure 11b(iii)).

**FIGURE 11:**
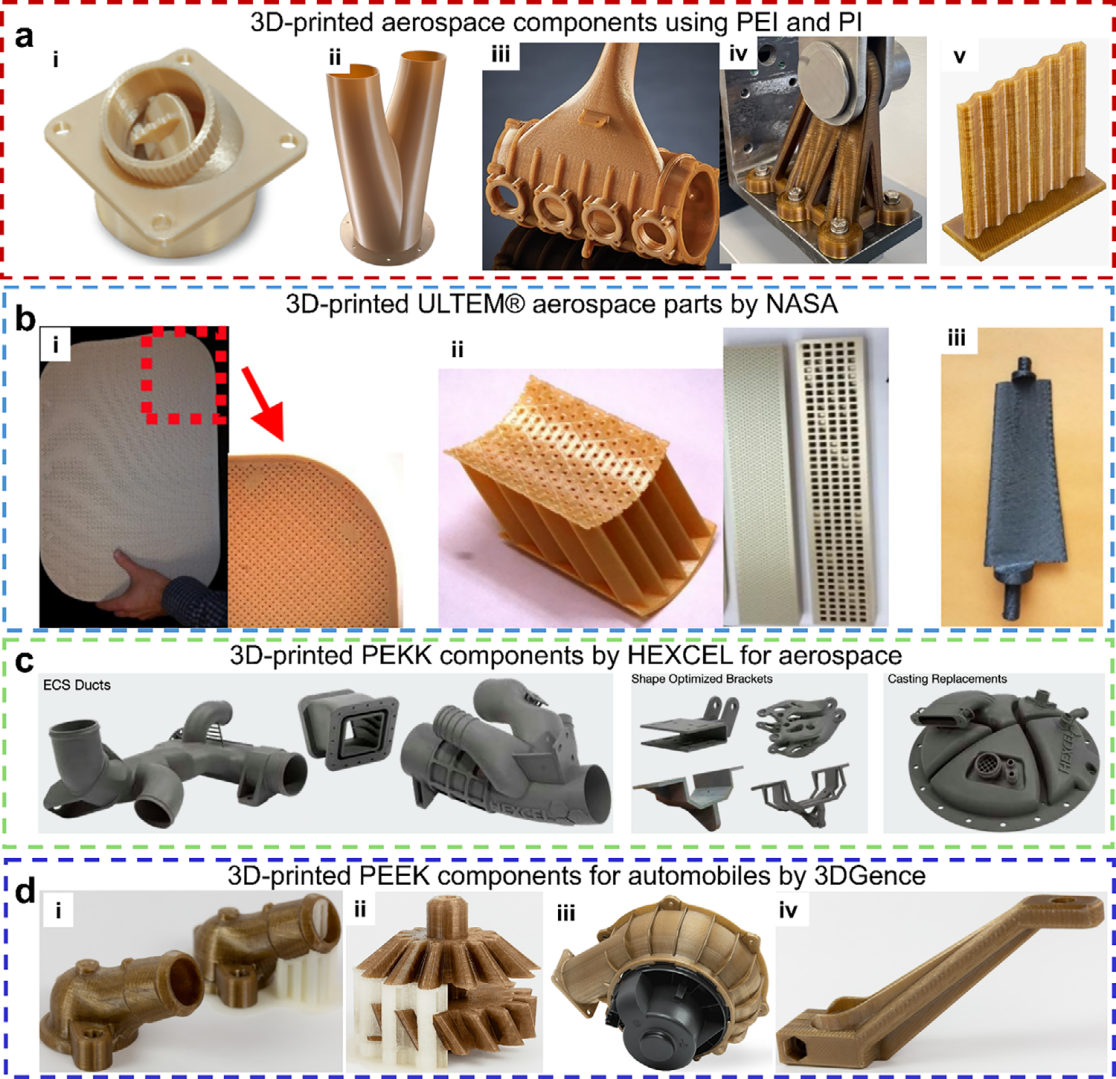
Additively manufactured high‐performance polymer components for high‐temperature applications with different industrial‐grade printing processes. (a) Parts printed with FFF process for aerospace applications using PEI and PI filaments: (i) Airbus A350 XWB components using ULTEM 9085 by Stratasys  [65]. Photo by courtesy of Stratasys, 2026, (ii) Aerospace Y‐duct using ULTEM 9085 by AON3D M2+ FFF printer  [23]. Photo by courtesy of AON3D, 2026, (iii) 3‑L V6 intake manifold using ULTEM 1010 by Stratasys [138]. Photo by courtesy of Stratasys, 2026, (iv) Aerospace bracket using PI by AON3D  [23]. Photo by courtesy of AON3D, 2026, and (v) FFF‐printed part using PI filament (EXTEM AMHH811F) by ROBOZE, which is suitable for sensor housing, avionics enclosure, and thermal and acoustic shielding  [139]. Photo by courtesy of Roboze, 2026. (b) FFF printed parts using ULTEM 9085 and ULTEM 1010 with 10% chopped carbon fiber (CF) by a NASA research center for space applications: (i) Perforated engine access door using ULTEM 9085, (ii) Acoustic liner and components using ULTEM 9085, and (iii) Composite vanes using ULTEM 1010 with 10% chopped CF [66]. Public use permitted. (c) Aerospace components printed with the FFF process using HexPEKK filament by Hexcel. Reproduced from ref. [20] under the terms of the Creative Commons Attribution (CC BY 4.0) license. (d) FFF‐printed parts using PEEK filament with an industrial‐grade 3D printer, 3DGence INDUSTRY F340, as the end‐use components for AGH Formula Student racing cars: (i) Connector housing, (ii) mechanical gears for drivetrain/steering systems, (iii) differential/actuator casing, and (iv) linkage arm [68]. Photo by courtesy of 3DGence, 2026.

Antero 800NA, a PEKK‐based FFF filament, is a suitable choice for avionic cabinets and aircraft interiors, where regulations regarding off‐gassing and structural integrity are crucial. This fulfills Boeing qualification (BMS8‐444), which attests to the reliable functioning of the system in operational settings. It was designed for Stratasys industrial‐grade FFF printers (F900 and Fortus 450mc), showcasing superior thermal and chemical resistance, mild elongation (6%), and high tensile strength (93 MPa) [140]. Its comparatively low elongation, however, can restrict its use in high‐strain or fatigue load scenarios, where more compliant materials are preferred. Similarly, HexPEKK EM, a conductive FFF filament from Hexcel, has been utilized for flight‐ready components (Figure 11c), which provides integrated radiation protection and electromagnetic shielding [20, 141]. In addition, this filament can also be used for spacecraft modules. These capabilities demonstrate the adaptability of 3D‐printed HPP PEKK parts for multifunctional designs through the use of additives or nanofillers. PEEK is also increasingly being utilized to replace metallic components in aeronautical electrical systems, including connector housings, gears, actuator casings, and linkage arms (Figure 11d), where thermal stability and corrosion resistance are crucial [68]. However, the extended durability of these 3D‐printed components under cyclic loads and thermal conditions, particularly at higher elevations, has yet to be extensively studied.

Another prominent example is the usage of Z2 PI, an industrial‐grade filament, designed for high‐temperature commercial printing systems like the AON3D M2+. In the aviation industry, this material was used for printing a load‐bearing support bracket (Figure 11a(iv)) for a freight plane. Tests of this bracket, conducted by the Royal Netherlands Navy's Expertise Center Additive Manufacturing (ECAM), revealed an outstanding tensile load‐bearing capability of 432 kg. The printed PI component exhibited an elevated degree of isotropy, which represents a notable improvement compared to ULTEM 9085, indicating enhanced interlayer adhesion. This isotropic characteristic is critical in aeronautical situations, where variable loading conditions necessitate consistent efficiency in all directions. The printed PI parts have also undergone FAR 25.853 certification, demonstrating their excellent inherent flame retardancy and suitability for high‐temperature aircraft interiors [23]. Moreover, ROBOZE has employed industrial‐grade PI filament, EXTEM AMHH811F produced by SABIC, using a commercial ARGO 500 FFF 3D printer. It has successfully printed this high‐performance filament for various aircraft components, including sensor housings, avionics enclosures, and thermal and acoustic shielding (Figure 11a(v)), demonstrating the suitability of PIs for multifunctional, demanding applications [67]. Overall, these applications demonstrate the potential of 3D‐printed HPPs to reduce manufacturing costs, weight, and part counts in the aerospace industry.

### 5.2 3D‐Printed HPPs in the Automotive Industry

In automotive applications, high thermal stability, chemical resistance, and mechanical strength under dynamic conditions make 3D‐printed HPPs such as PEI, PEEK, and PTFE suitable for optimized lightweight functional components. Thermal resistance is one of the key benefits of ULTEM 1010, which has been utilized with the FFF technique in the automotive industry to build an intake manifold for a V6 engine (Figure 11a(iii)) [138]. However, problems such as anisotropic shrinkage and residual stress must be addressed to achieve dimensional integrity under the elevated thermal and mechanical loads typical of engine bay environments. The automotive industry is also increasingly using PAEK‐based polymers, particularly PEEK and PEKK, as lightweight substitutes for metallic components due to their remarkable mechanical strength, chemical resistance, and thermal stability. Universities such as AGH University of Science and Technology in Kraków, Poland, have used the FFF technique with PEEK for printing different components of Formula Student vehicles, with an emphasis on boosting the power‐to‐weight ratio [68]. While manufacturability and reduced component weight are obvious benefits in the automotive industry, these applications are typically confined to non‐structural applications due to the difficulties of assuring strong interlayer bonding, good surface finish, and dimensional integrity in high‐speed conditions.

### 5.3 Other Applications of 3D‐Printed HPPs

Besides automotive and aerospace applications, AM of HPPs has also been extensively used in other promising sectors, including defense, marine, and radiation shielding applications. For instance, Feier et al. designed and 3D‐printed novel anti‐ballistic shields using PEI. The structures were able to withstand hypervelocity projectiles traveling at speeds of up to 6.5 km/s [142]. The 3D‐printed shields featured a Zig‐Zag baffle design that forced the debris cloud to follow a tortuous path, enhancing energy dispersion and impact resistance. On the other hand, for offshore applications, Wang et al. demonstrated the application of 3D‐printed HPPs in marine environments by focusing on graphene nanoplatelet and carbon fiber‐reinforced polyphenylene sulfide (GNP/CF‐PPS) composites [143]. The structures were subjected to high‐temperature aging tests to simulate harsh maritime conditions. At elevated temperatures (260°C), the material showed a 6.7% increase in stiffness and a 25.6% reduction in bending deflection. Furthermore, they found reduced porosity and tighter interlayer bonding in GNP‐modified samples, which contributed to enhanced structural integrity. They also demonstrated superior thermal stability with 7% higher crystallinity and lower mass loss during thermal degradation. These improvements, combined with the ability to maintain mechanical properties under prolonged high‐temperature exposure, highlight the potential of 3D‐printed GNP/CF‐PPS composites for reliable, long‐term service in marine structures and offshore components. Furthermore, for advanced radiation shielding applications, Wu et al. developed novel composites by combining tungsten reinforcements with a PEEK substrate. The new shielding materials demonstrated superior shielding of low‐energy ^137^Cs compared to high‐energy ^60^Co. The 3D‐printed PEEK‐tungsten composites also exhibited excellent mechanical properties after heat treatment [144]. These structures were designed to serve as gamma‐ray shielding elements in extreme environments such as space nuclear power systems, where lightweight yet high‐strength radiation shielding materials are critical for protecting astronauts and sensitive electronics. In another study, Cao et al. developed 3D‐printed PEEK composites for lightweight, multifunctional radiation shielding by integrating boron carbide (B_4_C) and tungsten (W) fillers [145]. The printed structures demonstrated efficient absorption of both neutrons and photons while maintaining superior mechanical strength. These developments highlight the capability of AM techniques in producing compact and durable shielding components, especially for nuclear facilities.

These examples establish technical feasibility across several sectors, but isolated component demonstrations do not by themselves establish qualification or deployment readiness. The following sections therefore benchmark AM of HPP systems against conventional alternatives and classify their maturity using evidence of process control, property retention, certification, and field deployment.

### 5.4 Competitive Benchmarking Strategy

Despite considerable progress in AM of HPPs, large‐scale industrial adoption depends on how effectively printed components can compete with metallic systems and conventionally manufactured engineering thermoplastics in terms of structural efficiency, durability, manufacturability, and long‐term service reliability. Therefore, direct benchmarking against traditional materials is essential for evaluating the true technological and deployment potential of 3D‐printed HPP systems under extreme operational conditions. Table 14 provides a qualitative comparison of 3D‐printed HPP structures with conventionally manufactured polymer and metallic systems. These synthesis‐level ratings are not universal rankings and depend on material grade, AM route, processing history, geometry, test method, and service environment.

**TABLE 14:** Qualitative performance benchmarking of additively manufactured high‐performance polymers.

Performance index	3D‐printed HPPs	Conventional manufacturing	Metallic systems	Engineering perspective
Design customization	Excellent	Limited	Limited	Broad AM advantage
Strength‐to‐weight efficiency	Excellent	Excellent	Moderate	Key advantage for automotive and aerospace applications
Chemical resistance	Excellent	Excellent	Often inferior	Strong advantage of HPPs
Thermal stability	Excellent	Excellent	Excellent	Suitable for many aerospace applications
Fatigue resistance	Moderate	Higher	Higher	Limited by porosity and interlayer defects
Certification maturity	Emerging	Established	Established	Key barrier for industrial adoption

### 5.5 Technology Readiness and Deployment Status

Although numerous 3D‐printed HPP systems have demonstrated promising laboratory‐scale performance, only a limited number have progressed toward industrial qualification and real‐world deployment. Readiness should therefore be assigned from demonstrated qualification or deployment evidence rather than from material capability alone. Table 15 summarizes representative systems according to the evidence reported in the reviewed literature and identifies the principal technical gaps that still limit broader deployment.

**TABLE 15:** Deployment readiness of additively manufactured high‐performance polymers.

Application	Representative HPPs	Status	Qualification evidence	Remaining challenge
Aerospace brackets/ducts	PEI (ULTEM 9085) [146]	Application‐specific deployment‐ready	FST (FAR 25.853) compliant; flight‐certified for Airbus A350 XWB.	Long‐term creep, fatigue, and heat exposure
Avionic cabinets	PEKK (Antero 800NA) [147]	Application‐specific deployment‐ready	Meets Boeing BMS8‐444 qualification for off‐gassing and integrity	Production cost and application‐specific structural validation
Radiation Shielding	PEEK/ Tungsten [144]	Laboratory‐scale/niche demonstration	Demonstrated shielding efficiency for space nuclear systems	Long‐term shielding performance retention
Superhydrophobic seals	PTFE‐based systems	Laboratory‐scale	Excellent water repellency	Long‐term durability and fatigue resistance

### 5.6 Maturity Classification for Additive Manufacturing of HPPs

Given the substantial variation in industrial readiness, process reliability, and long‐term performance across different HPP additive manufacturing systems, it is important to distinguish between fully validated technologies, emerging structural platforms, and early‐stage experimental approaches. The categories in Table 16 are evidence‐based maturity groupings derived from the systems discussed in this review; they should not be interpreted as universal technology‐readiness levels for an entire polymer family or AM route. This distinction provides a more realistic view of current deployment potential and of the validation needed for progression between tiers.

**TABLE 16:** Classification of maturity tiers for additive manufacturing of high‐performance polymers.

Maturity tier	Technology readiness	Representative HPPs	Preferred AM routes	Representative evidence	Analytical focus
Tier 1: Application‐specific mature and validated	Industrially deployed, application‐specific technologies	PEI (ULTEM 9085/1010), PEKK (Antero 800NA)	Industrial FFF	Flight‐certified aerospace components (e.g., Airbus A350 XWB); Boeing‐qualified (BMS8‐444) PEKK systems	Benchmarking structural equivalence and FST compliance
Tier 2: Emerging & structurally promising	Prototype and pre‐industrial structural systems	CF/GF‐reinforced PEEK and PEKK composites	FFF, SLS	Demonstrated use in AGH Formula Student racing cars and Boeing CST‐100 Starliner components	Managing the porosity‐toughness trade‐off and optimizing thermal gradients
Tier 3: Early stage/ experimental	Laboratory‐scale technologies	DIW of PI and PTFE architectures	DIW, UV‐DIW, FADIW	Laboratory‐scale demonstrations of self‐supporting springs, aerogels, and superhydrophobic structures	Overcoming massive dimensional shrinkage (∼45%) and rheological bottlenecks

## 6 Future Perspectives and Conclusions

The successful integration of AM techniques with HPPs is crucial for extreme environments and demanding applications. Despite substantial progress, industrial deployment remains limited by process variability, defect formation, insufficient long‐term durability data, and incomplete qualification pathways. Future research should therefore distinguish between near‐term priorities focused on process consistency and longer‐term opportunities aimed at developing intelligent, self‐monitoring, and adaptive HPP components.

### 6.1 Near‐Term Priorities: Addressing Process Consistency

The broader industrial adoption of 3D‐printed HPPs is limited not solely by the ability to print complex architectures, but by the challenge of maintaining geometric consistency, reproducibility, and reliable functionality. Throughout this review, we have identified several recurring bottlenecks in the AM of HPPs, including process variability, thermal history, porosity, interlayer defects, layer delamination, residual stresses, material anisotropy, and dimensional warpage. These factors act as key barriers to standardized qualification for target‐specific safe operations. Addressing these barriers is therefore crucial for achieving reliable structural functionality and qualification for safe operations.

In this regard, near‐term research efforts should emphasize improving process consistency and structural reliability rather than merely expanding printing capability. Among various advanced technologies, parametric optimization for improved consistency could be achieved more reliably using machine‐learning (ML)‐assisted approaches. This ML approach can facilitate the simultaneous optimization of multiple influential processing factors, including extrusion temperature, chamber temperature, print bed temperature, print speed, layer height, raster orientation, laser power intensity, and thermal history. Consequently, the process variability could be reduced substantially, thereby improving the consistency and reproducibility of the AM systems using HPPs [48, 86]. Such an ML‐based approach transforms the overall AM systems from empirical parameter selection to data‐driven optimization, which could enable fabrication techniques with improved consistency, reliability, and reproducibility.

Similarly, real‐time data acquisition and process monitoring are essential in maintaining process consistency. For instance, the integration of thermal imaging systems offers continuous monitoring and real‐time data acquisition of thermal history during the printing process, enabling the identification and control of the instantaneous thermal gradients. Such integration could substantially contribute to minimizing residual stress and warpage and thereby facilitating material diffusion for improved interlayer bonding in 3D‐printed HPPs [51]. Further integration with feedback and adaptive control systems can substantially improve process consistency, dimensional accuracy, repeatability, and reliable functionality.

Additionally, integrating a convolutional neural network (CNN)‐based algorithm into the AM route and process selection could be a smart, assistive quality‐assurance approach. As mentioned earlier, long‐term durability and fatigue performance of the 3D‐printed HPPs are primarily affected by incomplete material fusion, porosity, residual stress, air traps, and poor interlayer bonding. A CNN‐based defect‐identification approach enables real‐time image analysis, defect detection, and detection of incomplete material fusion. The real‐time feedback control systems can then use these signals to automatically control the various process parameters, including nozzle temperature, chamber temperature, print bed temperature, print speed, or laser energy intensity [128, 129, 130, 131]. Collectively, these integrated systems can pave the way for an effective and reliable closed‐loop fabrication approach that can detect, evaluate, and correct various inconsistencies, defects, and process variability, thereby improving the overall process consistency, reproducibility, and functional reliability for crucial high‐temperature applications. However, real‐time machine‐learning control requires rapid data acquisition, analysis, and communication between sensors and the printer. Where such computational demands remain impractical, multi‐fidelity physics‐informed models provide an alternative. Molecular dynamics, computational fluid dynamics, finite element analysis, and deep‐learning methods can be combined to predict thermal profiles, crystallization, interlayer diffusion, residual‐stress development, and defect formation. These models should also account for AM‐specific variables such as raster angle, layer thickness, and build orientation when predicting performance under extreme conditions.

### 6.2 Long‐Term Opportunities: Toward Self‐Monitoring and Self‐Healing Components

Once the process consistency is fully established through these near‐term approaches, future research should move progressively beyond process optimization. Long‐term approaches need to be developed for improving material durability, structural functionality, and applicability of the 3D‐printed HPPs in extreme environments. This suggests that future work should no longer be limited to fabricating defect‐free components; rather it must develop strategic AM routes and HPP systems that maintain structural viability and functionality, along with self‐healing characteristics, throughout their service in extreme environment applications. In this regard, the integration of localized in situ welding systems can play a crucial role in facilitating the interlayer diffusion kinetics through electromagnetic or induction heating, thereby reducing interlayer delamination and material anisotropy, and improving the overall structural functionality [21, 148]. Similarly, a structural health monitoring (SHM) technique can be incorporated into the 3D‐printed HPP structures. Such SHM systems can have embedded electronics and sensors for monitoring structural damage, fatigue, crack initiation, crack propagation, material deformation, and localized stress accumulation [127]. Based on real‐time feedback control systems, the localized in situ welding systems can be activated to repair the identified issues. Furthermore, an integrated self‐healing polymer system can play a crucial role in repairing the identified damage through thermally activated chain interdiffusion, supramolecular chemistry, or dynamic covalent interactions [149]. Collectively, these integrated advanced techniques can transform the existing systems into a more reliable and smarter system that can detect, evaluate, and repair the in situ structural defects, thereby enhancing the overall system's reliability and structural durability.

### 6.3 Bridging Qualification Gaps for Industrial Deployment

Despite the rapid advancement of additive manufacturing technologies for high‐performance polymers, several fundamental scientific and engineering challenges continue to impede their widespread deployment in mission‐critical applications. Addressing these unresolved issues is essential for achieving reliable, scalable, and certification‐ready HPP manufacturing platforms for extreme‐service environments. Table 17 presents an overview of four critical interconnected pillars, including materials, sensing, process control, and qualification, for future process development and requirements for target‐specific deployment of 3D‐printed HPPs. These pillars collectively define a strategic and practical roadmap to scale up the AM processes of HPPs for industrial and target‐specific high‐temperature applications.

**TABLE 17:** Major required technological advances and deployment targets for next‐generation additively manufactured high‐performance polymers.

Pillar	Required advances	Target deployment goal
Materials	Control reinforcement dispersion and interfaces, moisture resistance, and self‐healing/interlayer repair [15, 21, 148, 149]	Improve durability and damage tolerance
Sensing	Thermal imaging, CNN defect detection, and structural health monitoring [51, 127, 128, 130, 131]	Enable real‐time quality and health monitoring with maintenance decision‐making
Process control	ML optimization, closed‐loop control, and physics‐informed modeling [48, 86, 150, 151]	Improve consistency and reproducibility
Qualification	High‐temperature fatigue, creep, impact, and long‐term durability evaluation [152]	Support long‐term reliability certification

In summary, 3D‐printed HPPs hold immense potential for extreme‐environment applications due to their superior thermal, mechanical, and chemical stability. While advances in AM techniques, material modifications, and process optimizations have improved their performance, challenges remain in achieving consistent interlayer adhesion and predictive process–structure‐property control. Integrating emerging approaches such as machine learning‐guided process optimization, physics‐based simulations, and real‐time SHM systems could enable the 3D printing of intelligent, damage‐tolerant HPP structures. Continued research along these lines will be key to unlocking the full potential of HPPs for next‐generation high‐performance applications in extreme environments.

## Author Contributions

**Md Tofazzal Hossain**: writing – original draft, data curation, methodology, investigation. **Mohammed Intishar Nur**: writing – original draft, data curation, methodology, investigation. **Md Shajedul Hoque Thakur**: formal analysis, writing – review and editing, validation. **Indranil Dey**: data curation, investigation, writing – original draft. **Md Mesbahul Maruf**: data curation, investigation, writing – original draft. **Md Jahid Hasan**: data curation, investigation, writing – original draft. **Suryasarathi Bose**: writing – review and editing, validation. **Muhammad M. Rahman**: conceptualization, supervision, funding acquisition, writing – review and editing.

## Conflicts of Interest

The authors declare no conflicts of interest.

## Data Availability

The data that support the findings of this study are available from the corresponding author upon reasonable request.
